# Broadly neutralizing antibodies to SARS-related viruses can be readily induced in rhesus macaques

**DOI:** 10.1126/scitranslmed.abl9605

**Published:** 2022-08-10

**Authors:** Wan-ting He, Meng Yuan, Sean Callaghan, Rami Musharrafieh, Ge Song, Murillo Silva, Nathan Beutler, Wen-Hsin Lee, Peter Yong, Jonathan L. Torres, Mariane Melo, Panpan Zhou, Fangzhu Zhao, Xueyong Zhu, Linghang Peng, Deli Huang, Fabio Anzanello, James Ricketts, Mara Parren, Elijah Garcia, Melissa Ferguson, William Rinaldi, Stephen A. Rawlings, David Nemazee, Davey M. Smith, Bryan Briney, Yana Safonova, Thomas F. Rogers, Jennifer M. Dan, Zeli Zhang, Daniela Weiskopf, Alessandro Sette, Shane Crotty, Darrell J. Irvine, Andrew B. Ward, Ian A. Wilson, Dennis R. Burton, Raiees Andrabi

**Affiliations:** 1 Department of Immunology and Microbiology, The Scripps Research Institute, La Jolla, CA 92037, USA.; 2 IAVI Neutralizing Antibody Center, The Scripps Research Institute, La Jolla, CA 92037, USA.; 3 Consortium for HIV/AIDS Vaccine Development (CHAVD), The Scripps Research Institute, La Jolla, CA 92037, USA.; 4 Department of Integrative Structural and Computational Biology, The Scripps Research Institute, La Jolla, CA 92037, USA.; 5 Koch Institute for Integrative Cancer Research, Massachusetts Institute of Technology, Cambridge, MA 02139, USA.; 6 Alpha Genesis, Yemassee, SC 29945, USA.; 7 Division of Infectious Diseases and Global Public Health, Department of Medicine, University of California, San Diego (UCSD), La Jolla, CA 92037, USA.; 8 Computer Science and Engineering Department, University of California, San Diego (UCSD), La Jolla, CA 92037, USA.; 9 Center for Infectious Disease and Vaccine Research, La Jolla Institute for Immunology, La Jolla, CA 92037, USA.; 10 Department of Biological Engineering, Massachusetts Institute of Technology, Cambridge, MA 02139, USA.; 11 Howard Hughes Medical Institute, Chevy Chase, MD 20815, USA.; 12 Ragon Institute of Massachusetts General Hospital, Massachusetts Institute of Technology, and Harvard University, Cambridge, MA 02139, USA.; 13 Skaggs Institute for Chemical Biology, The Scripps Research Institute, La Jolla, CA 92037, USA.

## Abstract

To prepare for future coronavirus (CoV) pandemics, it is desirable to generate vaccines capable of eliciting broadly neutralizing antibody responses to CoVs. Here, we show that immunization of macaques with SARS-CoV-2 spike (S) protein with a two-shot protocol generated potent serum receptor binding domain cross-neutralizing antibody responses to both SARS-CoV-2 and SARS-CoV-1. Furthermore, responses were equally effective against most SARS-CoV-2 variants of concern (VOCs) and some were highly effective against Omicron. This result contrasts with human infection or many two-shot vaccination protocols where responses were typically more SARS-CoV-2 specific and where VOCs were less well neutralized. Structural studies showed that cloned macaque neutralizing antibodies, particularly using a given heavy chain germline gene, recognized a relatively conserved region proximal to the angiotensin converting enzyme 2 receptor binding site (RBS), whereas many frequently elicited human neutralizing antibodies targeted more variable epitopes overlapping the RBS. B cell repertoire differences between humans and macaques appeared to influence the vaccine response. The macaque neutralizing antibodies identified a pan-SARS-related virus epitope region less well targeted by human antibodies that could be exploited in rational vaccine design.

## INTRODUCTION

The rapid development of multiple vaccines to counter the coronavirus disease 2019 (COVID-19) pandemic caused by SARS-CoV-2 has been a triumph for science and medicine. The reason for these successes is likely due, in part, to the relative ease of inducing protective neutralizing antibodies (nAbs) in humans by immunization with the spike (S) protein, the key component of most vaccines (1–4). Different presentations of S-protein in the vaccines, particularly the mRNA-based vaccines, induce relatively high serum nAb responses in most individuals that appear to strongly target epitopes that overlap the angiotensin converting enzyme 2 (ACE2) receptor binding site (RBS) in the receptor binding domain (RBD) (1, 5–9). Infection with SARS-CoV-2 produces more variable nAb responses, but again, there is a strong targeting of the RBD and the RBS (1, 10–23).

A number of different antibody (Ab) germline genes are used in human nAbs to SARS-CoV-2, but a key contributor to the RBS response in many individuals is the human *VH3–53* Ab germline gene that encodes a subset (RBS-A, class 1) of highly potent immunodominant nAbs (22, 24, 25). Monoclonal Abs (mAbs) to the RBS that use *VH3–53* can be exceptionally potent, neutralizing with half maximal inhibitor concentration (IC_50_) values in the single-digit nanograms per milliliter range (10, 11, 22). These Abs typically show low degrees of somatic hypermutation (SHM); therefore, in a sense, the human naïve Ab repertoire is well suited to responding to SARS-CoV-2 with nAbs. However, there is notable sequence variation in the RBS between sarbecoviruses, for example, between SARS-CoV-2 and SARS-CoV-1, despite the fact that the viruses both use ACE2 as receptor. Some of the variation occurs in critical residues for *VH3–53*–mediated recognition, and nAbs using the *VH3–53* germline gene generally show very weak or no neutralization of SARS-CoV-1. Furthermore, many of the mutations found in variants of concern (VOCs) (26–30) are in the RBS and likely arise from the selection pressure of immunodominant RBS-directed nAbs (28, 30, 31).

We aimed to determine how nAb responses to the S-protein might arise in nonhuman primates (NHPs), knowing that differences exist between the naïve Ab repertoires of humans and rhesus macaques. When we launched the study, the mRNA vaccine immunogens used in humans were not available to us, but nevertheless, we considered it valuable to probe the S-protein–specific Ab response in NHPs. We immunized macaques with SARS-CoV-2 S-protein using a protocol that we have successfully used in HIV envelope protein immunization of NHPs (32) and investigated nAb responses with a particular interest in the neutralization breadth of such responses against sarbecoviruses.

## RESULTS

### SARS-CoV-2 S-protein prime-boost immunization leads to robust sarbecovirus broadly nAb responses in rhesus macaques

**Fig. 1.**
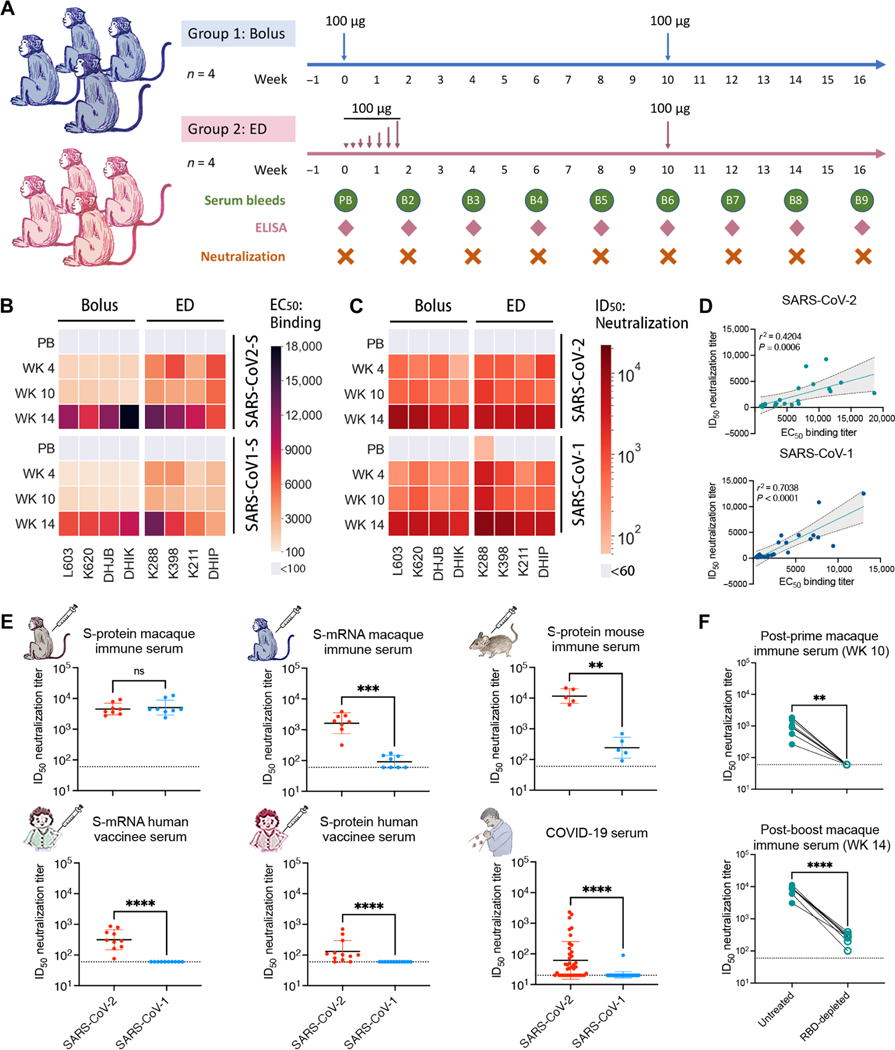
SARS-CoV-2 and SARS-CoV-1 cross-reactive antibody binding and neutralizing responses were observed in SARS-CoV-2 S-protein–immunized rhesus macaques. (**A**) The SARS-CoV-2 S-protein prime-boost immunization in rhesus macaques and sampling schedule is shown. Animals were primed at week 0 with 100 μg of SARS-CoV-2 S-protein along with adjuvant SMNP, by bolus (group 1: *n* = 4) or escalating dose (ED) (group 2: *n* = 4) immunization, and both groups further boosted at week 10 with 100 μg of the S-protein immunogen. Serum and peripheral blood mononuclear cells (PBMCs) were collected every 2 weeks for immune response analysis. (**B**) The heatmap shows EC_50_ ELISA binding of SARS-CoV-2 S-protein–immunized NHP serum from bolus and ED schemes against different S-proteins. EC_50_ binding responses for prebleed (PB), post-prime (WK 4 and WK 10), and post-boost (WK 14) sample time points are shown. (**C**) SARS-CoV-2– and SARS-CoV-1–specific serum ID_50_ neutralizing antibody titers were measured in SARS-CoV-2 S-protein–immunized rhesus macaques. ID_50_ neutralizing antibody responses are shown for PB, post-prime (WK 4 and WK 10), and post-boost (WK 14) sample time points. (**D**) NHP immune serum binding to SARS-CoV-2 or SARS-CoV-1 S-proteins correlated modestly with neutralization against the corresponding virus. SARS-CoV-2 or SARS-CoV-1 EC_50_ serum antibody binding titers and ID_50_ nAb titers were compared by nonparametric Spearman correlation two-tailed test with 95% confidence interval. The Spearman correlation coefficient *r* and the *P* value are indicated. (**E**) A comparison of cross-neutralizing activities is shown for SARS-CoV-2 S-protein–immunized NHP immune serum (week 14, after two immunizations at weeks 0 and 10), serum from S-mRNA–vaccinated NHPs, serum from S-mRNA–vaccinated humans, serum from S-protein–vaccinated human, serum from COVID-19–recovered humans, and serum from S-protein–immunized mice. Horizontal bars indicate geometric mean. Dashed horizontal lines indicate limit of detection. Statistical comparisons between groups were performed using a Mann-Whitney two-tailed test. **P < 0.01; ***P < 0.001; ****P < 0.0001; not significant (ns), *P* > 0.05. (**F**) Neutralization of SARS-CoV-2 by post-prime (WK 10) and post-boost (WK 14) macaque immune serum and the corresponding anti-RBD antibody–depleted serum (RBD-depleted) is shown. Anti-RBD polyclonal antibodies in the immune serum were depleted by adsorption on recombinant monomeric RBD, and the depleted serum was tested for SARS-CoV-2 neutralization. The SARS-CoV-2 ID_50_ neutralizing antibody titers for untreated and RBD-depleted serum were compared by Mann-Whitney two-tailed test; **P < 0.01 and ****P < 0.0001.

A recombinant prefusion-stabilized soluble S-protein plus a saponin (SMNP) adjuvant was administered through subcutaneous injection in naïve rhesus macaques (*n* = 8). Because the manner of immunogen administration may shape immune responses (32–34), two protocols were evaluated: a conventional prime-boost consisting of bolus injections (100 μg) at weeks 0 and 10 for group 1 animals (*n* = 4) and an escalating dose (ED) prime and bolus boost for group 2 (*n* = 4; Fig. 1A). Serum Ab responses to SARS-CoV-2 S-protein, evaluated by enzyme-linked immunosorbent assay (ELISA) (figs. S1 and S2), were detected in both groups at week 2, and, although responses at week 4 were stronger for the ED group, no major differences were found subsequently. Boost immunization at week 10 increased EC_50_ (half maximal effective concentration) binding titers to the 10^3^ to 10^4^ range for all animals, indicating a strong Ab recall response. ID_50_ (50% inhibitory dose) neutralization titers showed a similar pattern, where both immunization groups developed specific nAb responses by week 4 after boost, which were enhanced by week 12 after boost (figs. S1 and S2).

Next, we examined serum responses for cross-reactivity with SARS-CoV-1 (Fig. 1B and fig. S2). Strong cross-reactive binding responses to SARS-CoV-1 S-protein were observed (Fig. 1B and fig. S2), as well as strong cross-neutralizing Ab responses against pseudotyped SARS-CoV-1 (Fig. 1C and fig. S2). ID_50_ neutralization titers showed a modest correlation with EC_50_ binding titers for both pseudotyped SARS-CoV-2 and SARS-CoV-1 (Fig. 1D). The elicitation of potent serum cross-neutralizing responses in rhesus macaques by S-protein immunization is in stark contrast to human SARS-CoV-2 natural infection, which typically results in autologous nAb responses; cross-neutralizing activity against SARS-CoV-1 has been shown to be relatively rare in SARS-CoV-2 infection or vaccination (Fig. 1E and fig. S3) (35–37). In humans vaccinated twice with mRNA encoding S-protein, we similarly found SARS-CoV-2 autologous but not SARS-CoV-1 cross-reactive serum nAb responses (Fig. 1E and fig. S3) as also described by others (38). In humans vaccinated with the S-protein vaccine, serum samples showed no evidence of cross-neutralization of SARS-CoV-1 (Fig. 1E). In macaques, mRNA S-protein vaccination did produce some evidence of SARS-CoV-1 neutralization, but it was much weaker than that observed in our macaque immunization experiments. Thus, vaccination protocol and species may contribute to induction of serum cross-neutralizing responses to SARS-CoV-1 and SARS-CoV-2.

To further examine the role of species-specific B cell immunogenetic differences, we vaccinated a group of five mice twice (week 0, prime; week 3, boost) with SARS-CoV-2 S-protein and SMNP adjuvant (fig. S4). Consistent with earlier studies (39–41), SARS-CoV-2 S-protein immunization of mice elicited high titers of autologous SARS-CoV-2 nAbs; however, the SARS-CoV-1 cross-neutralizing Ab titers were substantially less than the macaque cross neutralizing Ab responses (Fig. 1E and fig. S4), indicating a strong species-dependent contribution to development of broadly nAbs (bnAbs) using SARS-CoV-2 S-protein as immunogen.

### Polyclonal macaque bnAb responses are directed to the RBD and are effective against diverse sarbecoviruses and SARS-CoV-2 VOCs

We next characterized polyclonal nAb specificities generated in the macaques by measuring the binding of immune serum in the presence of three well-characterized human nAbs to SARS-CoV-2 S-protein and RBD by Bio-layer Interferometry (BLI) (fig. S5). These nAbs define unique epitopes on the RBD and were isolated from a COVID-19 convalescent donor (CC12.3 and CC12.19) (11, 22) and a SARS-CoV-1 donor (CR3022) (11, 42). CC12.3 is a *IGHV3–53* nAb targeting the ACE2 binding site and designated as RBS-A or class 1 (22, 24, 28). CC12.19 recognizes a complex RBD epitope but competes with some non-RBD Abs (11). CR3022 recognizes the class 4 epitope site (24, 28). Macaque serum samples bound well to S-protein in the presence of the nAbs, suggesting that the RBD nAb response was a minor part of the total S-protein–specific response (fig. S5). However, binding to RBD was strongly inhibited at week 10 by all three nAbs, consistent with a strong response to RBD neutralizing epitopes (fig. S5). The inhibition was reduced after boosting, suggesting that Ab responses to B cell epitopes outside of the RBD may increase after secondary immunization (43, 44).

To further determine the contributions of RBD-specific Abs in polyclonal immune serum, we used recombinant RBD to deplete serum samples of RBD Abs and assessed neutralization at week 10 (post-prime) and week 14 (post-boost). Depletion eliminated neutralization by week 10 serum and greatly reduced activity of week 14 serum (Fig. 1F). Depletion of anti-RBD Abs was confirmed by BLI binding studies with SARS-CoV-2 RBD and S-protein (fig. S6). The results suggest that SARS-CoV-2 neutralization in both post-prime and post-boost macaque immune serum is dominated by RBD-specific nAb responses, but post-boost nAbs to non-RBD S-protein epitopes or trimer-dependent RBD epitopes may minimally contribute to neutralization.

**Fig. 2.**
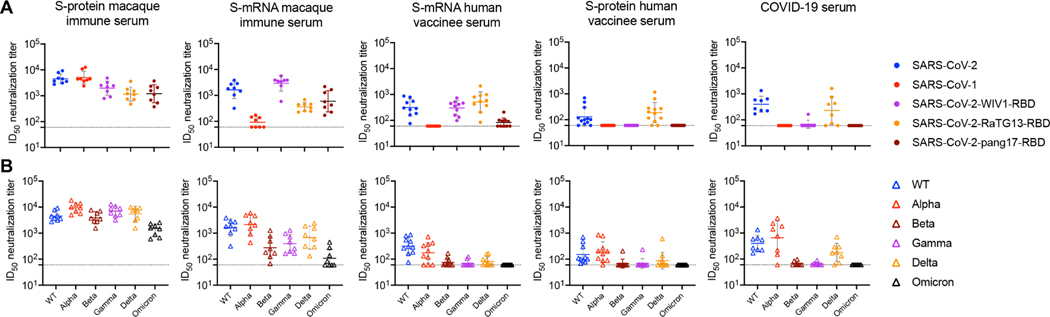
SARS-CoV-2 S-protein–immunized macaque serum samples show neutralization with sarbecovirus chimeric SARS-CoV-2 pseudotyped viruses and SARS-CoV-2 variants. (**A**) The dot plots show ID_50_ neutralizing antibody titers of SARS-CoV-2 S-protein–immunized week 14 macaque immune serum, S-mRNA macaque immune serum, S-mRNA human vaccinee serum samples, S-protein human vaccinee serum samples, and COVID-19 convalescent serum samples with SARS-CoV-2, SARS-CoV-1, and RBD-swapped WIV1, RaTG13, and pang17 chimeric SARS-CoV-2 pseudoviruses. (**B**) The dot plots show ID_50_ neutralizing antibody titers of the same serum samples from (A) with SARS-CoV-2 WT (Wuhan strain) and VOCs that have circulated in humans (Alpha, Beta, Gamma, Delta, and Omicron). For (A) and (B), ID_50_ neutralization titers less than 60 are shown as 60, and the dashed lines at 60 indicate the lower limit of detection. Horizontal bars indicate geometric mean values.

Because S-protein macaque immune serum competed strongly with human RBD nAbs and neutralization was largely RBD-directed, we investigated other sarbecoviruses by engrafting the RBD from those viruses onto the SARS-CoV-2 backbone (45–47). We chose the three sarbecoviruses, WIV1, RaTG13, and pang17, which use ACE2 receptor for cell entry and are phylogenetically related to SARS-CoV-2 and SARS-CoV-1 (Fig. 2A and fig. S7). The week 14 S-protein macaque immune serum samples consistently and potently neutralized all three chimeric pseudoviruses, with the WIV1 chimera being most effectively neutralized when averaged over all animals. Macaques given the mRNA vaccine, although showing relatively weak neutralization of SARS-CoV-1, do show consistent neutralization of the three chimeric pseudoviruses at concentrations comparable to those of S-protein–immunized animals. COVID-19 and S-protein vaccine serum samples only neutralized the closely related RaTG13 chimera at a lower concentration than the macaque serum samples. S-mRNA human immune serum samples showed moderate neutralization against WIV1 and RaTG13 chimeras and sporadic neutralization of the pang17 chimera. The results suggest the macaque immune serum targets more conserved RBD regions on sarbecoviruses than serum from human infection or human S-mRNA vaccination. The fine details of this cross-reactivity are consistent with the relatedness of these viruses to SARS-CoV-1 and SARS-CoV-2. RaTG13 RBD is closer to SARS-CoV-2 (fig. S7) and is neutralized more efficiently by COVID-19 serum samples, in which neutralization is predominantly RBD-directed. WIV1 is closer to SARS-CoV-1 and is neutralized more potently by the macaque immune serum that neutralizes SARS-CoV-1 more effectively than SARS-CoV-2.

Next, we compared the macaque and human serum samples for neutralization of SARS-CoV-2 incorporating key mutations from current SARS-CoV-2 VOCs, B.1.1.7 (Alpha), B.1.351 (Beta), P.1 (Gamma), B.1.617.2 (Delta), and B.1.1.529 (Omicron) (Fig. 2B) (9, 26, 28, 30). Neutralizing activities against Alpha were similar to those against the ancestral [wild-type (WT)] virus. The human COVID-19 infection, mRNA vaccine, and S-protein vaccine serum samples showed essentially complete loss of neutralizing activity against Beta, Gamma, and Omicron with some retained activity against Delta. Macaque S-protein and mRNA vaccine serum samples retained a notable degree of neutralizing activity against all tested VOCs. The S-protein–immunized macaques maintained relatively high serum neutralizing titers against Omicron. This result is again consistent with macaque serum samples targeting more conserved regions of the S-protein and thereby yielding Abs with less sensitivity to known escape mutations.

### mAbs from immunized macaques are potent and broadly neutralizing

**Fig. 3.**
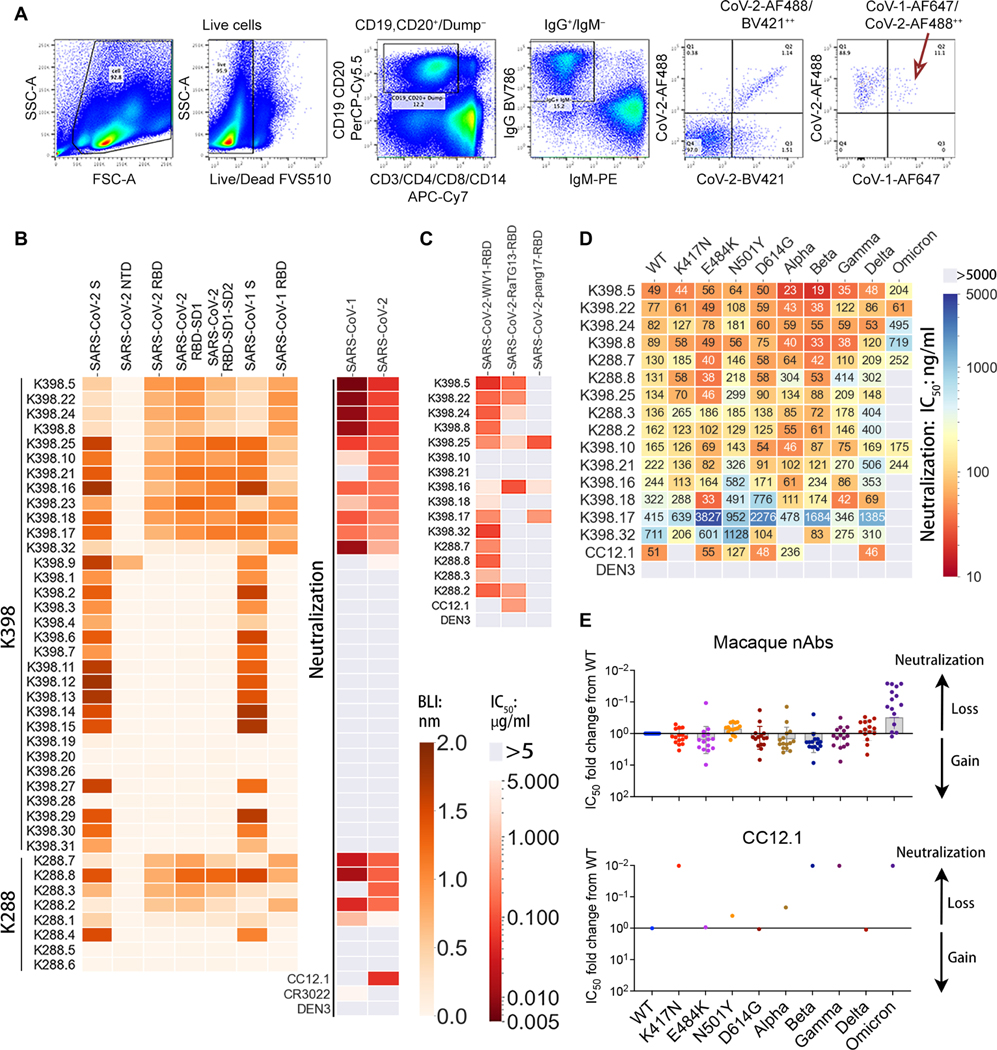
Isolation and characterization of cross-neutralizing monoclonal antibodies from two SARS-CoV-2 S-protein–immunized rhesus macaques. (**A**) The flow cytometry plots show the sorting strategy used to isolate single isotype-switched B cells that specifically bind to fluorescently labeled SARS-CoV-1 and SARS-CoV-2 S-protein probes. The class-switched memory B cells were gated as single, scattered, live (SSL), CD3^−^, CD4^−^, CD8^−^, CD14^−^, CD19^+^/CD20^+^, IgM^−^, and IgG^+^. The antigen-specific IgG^+^ B cells selected, as indicated by a red arrow, were used to obtain paired heavy and light chain immunoglobulin sequences for mAb expression and characterization. mAbs in this study were only isolated from S-protein–vaccinated rhesus macaques. (**B**) The heatmap shows the BLI binding responses of isolated monoclonal antibodies from rhesus macaques (K398 and K288) against SARS-CoV-2 S-protein, SARS-CoV-2 S-protein–derived domains and subdomains (NTD, RBD, RBD-SD1, and RBD-SD1–2), SARS-CoV-1 S-protein, and SARS-CoV-1-RBD (indicated with a green color scale). The IC_50_ neutralizing titer against SARS-CoV-1 and SARS-CoV-2 pseudoviruses is shown in the right columns with a red color scale. (**C**) Neutralization of SARS-CoV-2 RBD–swapped pseudoviruses by macaque cross-neutralizing mAbs are shown. IC_50_ values are color-coded, and an IC_50_ greater than 5 μg/ml is indicated in gray. (**D**) IC_50_ values are shown for macaque cross-neutralizing mAbs against pseudoviruses with single amino acid mutations in the SARS-CoV-2 S-protein and variants of concern (Alpha, Beta, Gamma, Delta, and Omicron). IC_50_ values greater than 5000 ng/ml are shown in gray. A dengue virus–specific antibody, DEN3, was used as a negative control in (B) to (D). (**E**) Neutralization IC_50_ fold changes of mAbs (*n* = 15) with mutants compared with the WT SARS-CoV-2 pseudovirus are shown in the dot plots with geometric mean labeled by the bar in gray. The gain or loss of neutralizing potency is indicated by the arrows on the right.

We isolated mAbs from two animals (K288 and K398) by sorting antigen-specific single B cells using SARS-CoV-2 and SARS-CoV-1 S-proteins as baits (Fig. 3A). Briefly, CD19^+^CD20^+^ immunoglobulin G–positive (IgG^+^) IgM^−^ B cells that recognized SARS-CoV-2 and SARS-CoV-1 S-protein were sorted from peripheral blood mono-nuclear cells (PBMCs) from immunized animals. Paired heavy and light chain sequences from single B cells were recovered, and 40 mAbs were expressed (Fig. 3B). BLI binding revealed that all but six mAbs exhibited binding to SARS-CoV-2 or SARS-CoV-1 S-protein (Fig. 3B and fig. S8). Fourteen of 34 mAbs showed cross-neutralization of SARS-CoV-1 and SARS-CoV-2, whereas 3 mAbs neutralized only SARS-CoV-2 (Fig. 3B and fig. S8). Unexpectedly, most cross-neutralizing mAbs were more potent against SARS-CoV-1 than SARS-CoV-2 (Fig. 3B and fig. S8). Seventeen of 34 mAbs bound S-protein only and not RBD or NTD (amino-terminal domain), but none showed neutralization. All neutralizing mAbs (except one NTD-specific mAb) bound strongly to RBDs (Fig. 3B and fig. S8). Overall, these results are consistent with the polyclonal nAb data described above (Fig. 1) and show that macaque immunization, here with S-protein, generates potent SARS-CoV-1 and SARS-CoV-2 nAb responses.

We next extended the neutralization studies to a range of sarbe-covirus RBD chimeric SARS-CoV-2 pseudoviruses (Fig. 3C and fig. S8). Most of the mAbs that showed potent SARS-CoV-1 and SARS-CoV-2 neutralization also neutralized WIV1, but activity against RaTG13 and pang17 was more sporadic. Some mAbs did neutralize all five sarbecoviruses tested, albeit with relatively lower neutralization potency (Fig. 3C and fig. S8). The cross-neutralization of mAbs was also consistent with BLI binding studies using monomeric RBDs derived from WIV1, RaTG13, and pang17 sarbecoviruses (fig. S9).

We also tested neutralization by these cross-reactive mAbs against SARS-CoV-2 VOCs and key mutants. Consistent with the macaque serum mapping above, the mAbs were roughly equally effective against variants Alpha, Beta, Gamma, and Delta with some Abs moderately more and some moderately less potent than against the ancestral virus (Fig. 3, D and E). For Omicron, most of the Abs were less potent (10- to more than100-fold), but about a third were approximately equally potent as against the ancestral virus. As a control, the RBS-A/class 1 nAb CC12.1 isolated from a COVID-19 human donor showed complete loss of neutralizing activity against Beta, Gamma, and Omicron variants (Fig. 3, D and E).

### bnAbs show a strong enrichment for the *IGHV3–73* heavy chain gene

**Fig. 4.**
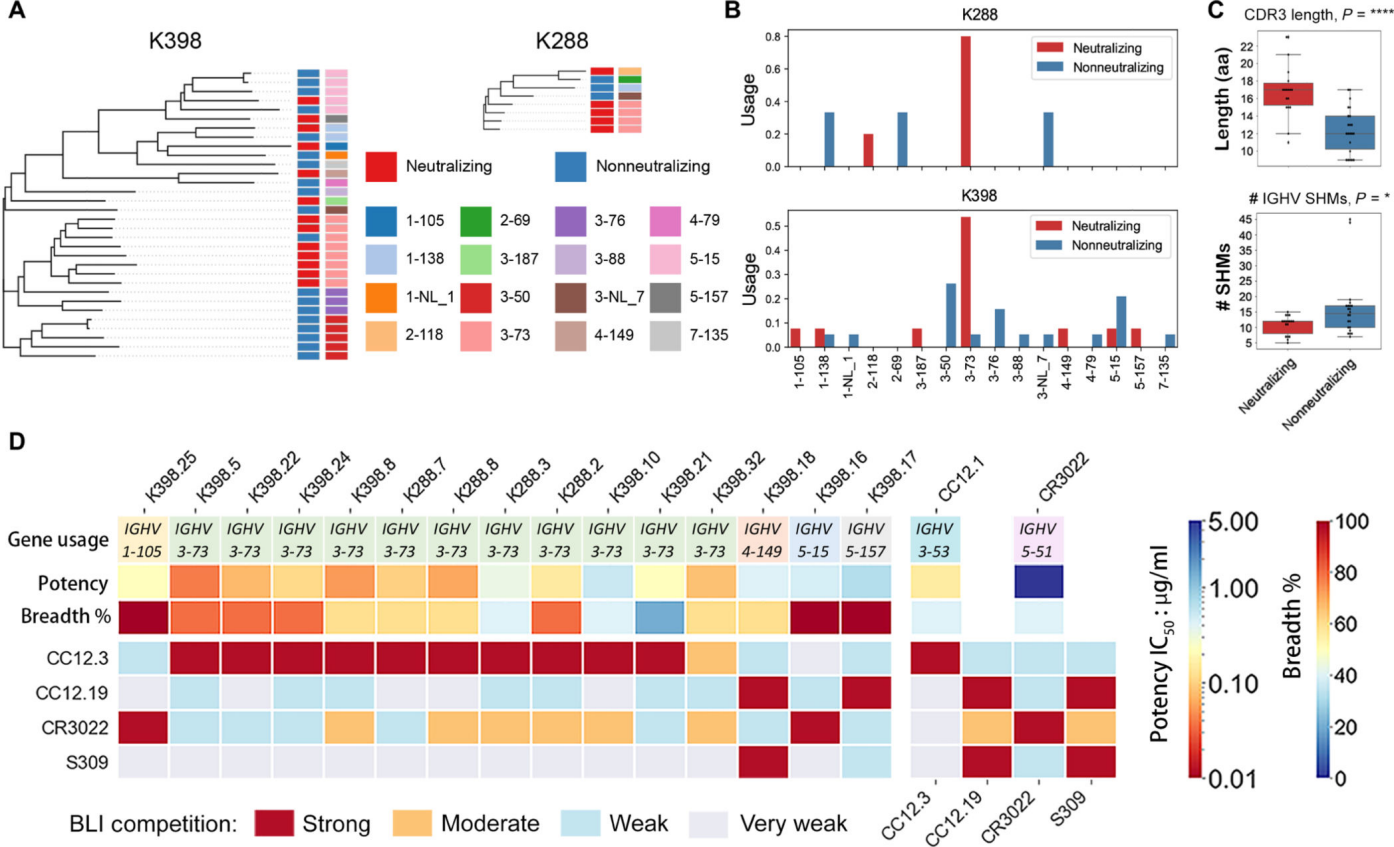
Immunogenetic and epitope properties of sarbecovirus cross-neutralizing macaque antibodies. (**A**) Phylogenetic trees derived from heavy chain (VH)–gene sequences of S-protein–specific antibodies isolated from K398 and K288 macaques are shown. Gene assignments used the rhesus macaque (*M. mulatta*) germline database described in (59). The *IGHV* gene usage for each mAb depicted in the tree is shown by different colors indicated in the bottom of the color scheme. Neutralizing and nonneutralizing mAbs are shown in red and blue, respectively. (**B**) *IGHV* gene usage for neutralizing and nonneutralizing mAbs in K288 and K398 macaques are shown. (**C**) CDRH3 length in amino acids (aa) and number of SHMs were quantified for neutralizing and nonneutralizing mAbs. *P < 0.1 and ****P < 0.0001. (**D**) The heatmap shows BLI competition–based epitope binning of macaque cross-neutralizing mAbs with human RBD-specific nAbs CC12.3, CC12.19, CR3022, and S309. The gene usage, geometric mean neutralization potency, and breadth [calculated from neutralization with five viruses in Fig. 3 (B and C)] for each nAb are indicated. The BLI competition experiment was performed with SARS-CoV-2 RBD, and the degree of competition is indicated as red (strong), orange (moderate), light blue (weak), and gray (very weak competition).

We next sought to determine properties of the macaque mAbs that would explain their broad neutralization. First, we analyzed the mAb sequences and found that they used a range of germline gene families with strong enrichment for *IGHV3–73* and, to some extent, *IGHV3–50* and *IGHV5–15*, heavy chain germline genes (Fig. 4A and fig. S10). None of the *IGHV3–73* Abs from either animal (K288 and K398) were clonal members, suggesting independent selection of this gene family with common VH-germline gene features (fig. S10).

The rhesus macaque *IGHV3–73* germline gene is closely related in sequence to human *IGHV3–53* and *IGHV3–66* genes, commonly used by human SARS-CoV-2 nAbs isolated from natural infection and vaccination, which initially suggested the possibility of convergent modes of RBD epitope recognition (fig. S11) (9, 20, 22, 25, 28). Some cross-neutralizing Abs were encoded by non–*IGHV3–73* gene families (Fig. 4, A and B, and fig. S10). Most of the *IGHV3–50* and *IGHV5–15* encoded Abs were nonneutralizing, including some clonally expanded Ab lineages isolated from animal K398 (Fig. 4, A and B, and fig. S10). CDRH3 (third complementarity determining region of Ab heavy chain) lengths ranged from 9 to 23 amino acid residues (Fig. 4C and fig. S10) with nAbs having relatively longer CDRH3s (median of 17 amino acids) than non-nAbs (median of 12 amino acids). Two nAbs used the longest CDRH3 of 23 amino acid residues (fig. S10). The nAbs had relatively lower SHM compared to non-nAbs (median number of nucleotide SHMs; neutralizing = 10, nonneutralizing = 14.5) (Fig. 4C and fig. S10).

We next mapped the epitopes recognized by the macaque RBD nAbs by BLI competition with human mAbs, CC12.3, CC12.19, CR3022, and S309. The specificities of CC12.3, CC12.19, and CR3022 are described above; S309 recognizes a unique epitope site, referred to as the class 3 epitope site (24, 28). All 11 macaque *IGHV3–73*–encoded SARS-CoV-1 and SARS-CoV-2 nAbs strongly competed with *IGHV3–53*–encoded CC12.3 nAb, and many showed moderate to weak competition with CR3022 (Fig. 4D and fig. S12). Two nAbs, K398.17 and K398.18, strongly competed with CC12.19 nAb, with K398.18 Ab also competing with S309 (Fig. 4D and fig. S12) (48). Two nAbs, K398.25 and K398.16, competed strongly with CR3022 that targets the class 4 site (Fig. 4D and fig. S12). The mAbs showing the greatest overall pan-sarbecovirus cross-neutralization breadth were those competing with CR3022 or CC12.19.

### Potent *IGHV3–73* bnAbs recognize a relatively conserved region on the RBD overlapping the RBS

**Fig. 5.**
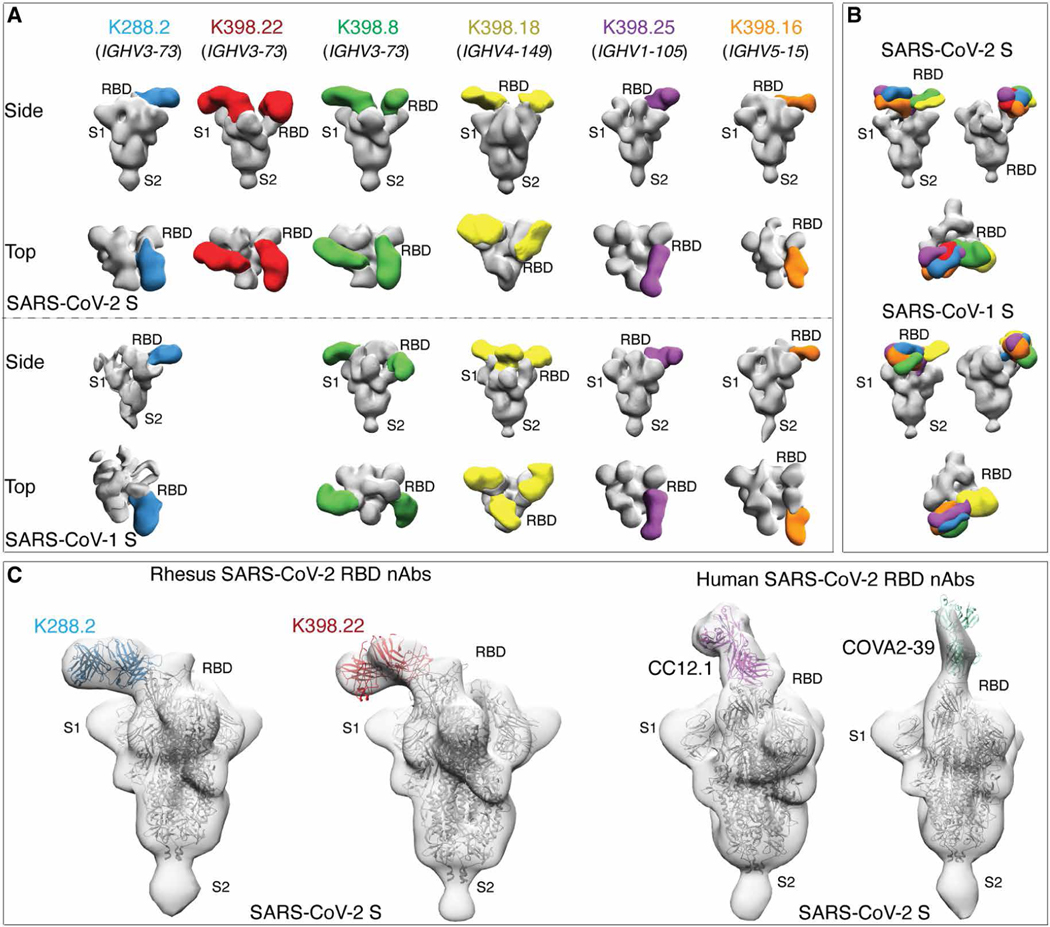
Electron microscopy structures of sarbecovirus cross-neutralizing macaque mAbs with SARS-CoV-2 and SARS-CoV-1 S-proteins. (**A**) Electron microscopy 3D reconstructions of rhesus macaque nAb Fabs with SARS-CoV-2 and SARS-CoV-1 S-proteins are shown. Fabs of six rhesus macaque nAbs, K288.2 (blue), K398.22 (red), K398.8 (green), (K398.18 (yellow), K398.25 (purple), and K398.16 (orange) were complexed with SARS-CoV-2 and SARS-CoV-1 S-proteins, and 3D reconstructions were generated from 2D class averages. The heavy chain germline gene usage is indicated for each macaque nAb. The S-protein S1 and S2 subunits and the RBD sites are labeled. (**B**) Side and top views showing 3D reconstructions of all six RBD-directed macaque cross-neutralizing Abs bound to SARS-CoV-2 (top) and SARS-CoV-1 (bottom) S-proteins. (**C**) S-protein binding angles of approach were compared between macaque *IGHV3–73*–encoded RBD cross nAbs, K288.2 and K398.22, and human *IGHV3–53*–encoded RBD nAbs, CC12.1 and COVA2–39, isolated from COVID-19 donors. The crystal structures of the respective nAbs were docked into EM density maps. The RBD, S1, and S2 subunits of SARS-CoV-2 S-protein are labeled. The macaque nAbs approach S-protein RBD from the side, and the two human nAbs approach at a more perpendicular angle close to the S-protein threefold axis.

We turned next to structural studies to gain a better understanding of neutralizing cross-reactivity. Single-particle, negative-stain electron microscopy (nsEM) was used to image representative macaque nAb Fabs, K288.2, K398.22, K398.8 (*IGHV3–73*), K398.18 (*IGHV4–149*), K398.25 (*IGHV1–105*), and K398.16 (*IGHV5–15*) with SARS-CoV-1 or SARS-CoV-2 S-proteins. NsEM confirmed binding of all six nAbs to S-protein RBD, where the cross-neutralizing Abs encoded by various germline V-genes interacted with the RBD through distinct binding modes (Fig. 5A and fig. S13). The binding modes were largely similar for SARS-CoV-2 and SARS-CoV-1 S-proteins, with some exceptions (Fig. 5, A and B, and fig. S13). We compared reconstructions of two macaque *IGVH3–73* and two human *IGVH3–53* nAbs and noted differences in the angles of approach to the SARS-CoV-2 RBD; the macaque cross-neutralizing Abs interacted with the RBD from a more lateral angle compared to the two human nAbs, which had a more perpendicular approach angle close to the S-protein threefold axis (Fig. 5C).

**Fig. 6.**
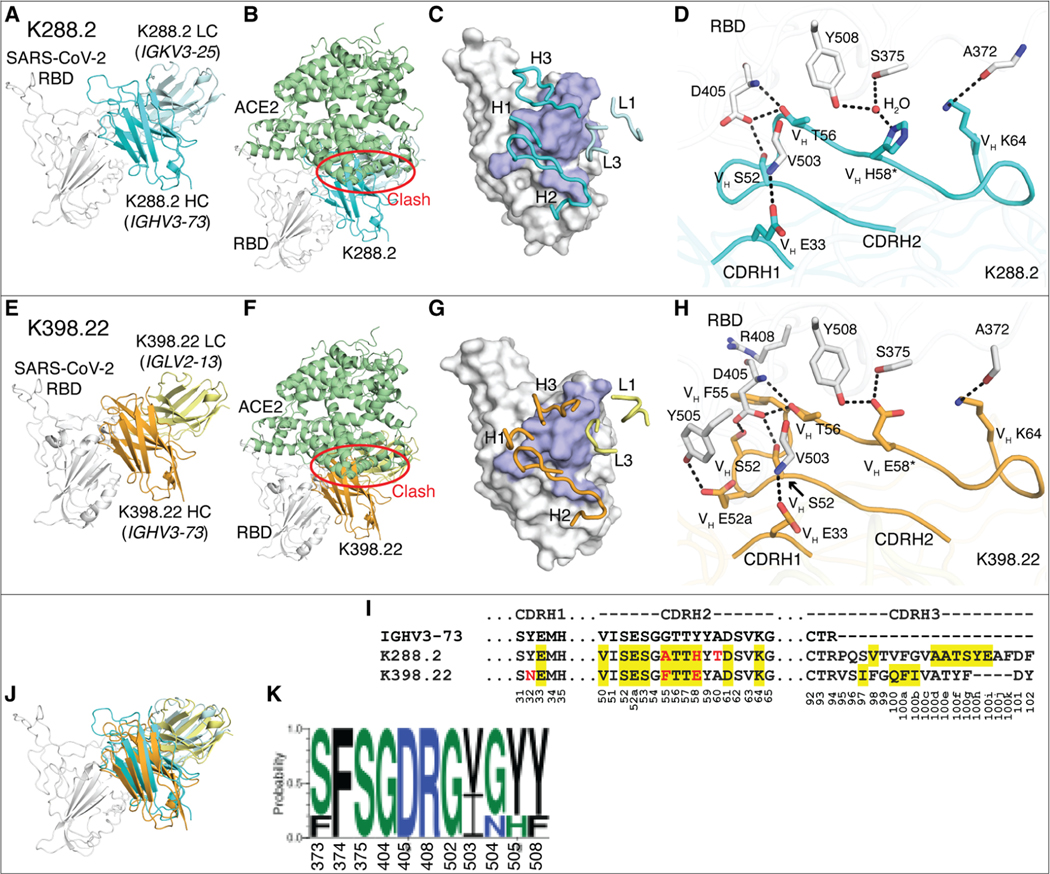
Crystal structures of two rhesus macaque *IGHV3–73*–encoded neutralizing antibodies bound to SARS-CoV-2 RBD. The SARS-CoV-2 RBD is shown in white throughout the figure and human ACE2 (hACE2) is shown in pale green. The heavy (HC) and light chains (LC) of K288.2 are in cyan and light cyan, and those of K398.22 are in orange and yellow. For clarity, only variable domains of the antibodies are shown. (**A** to **H**) Structures of SARS-CoV-2 RBD in complex with K288.2 antibody (A to D) and in complex with K398.22 antibody (E to H) are shown. (A and E) Germline genes encoding K288.2 (A) and K398.22 (D) macaque antibodies are labeled. (B and F) Structures of SARS-CoV-2 RBD in complex with K288.2 (B) or K398.22 (F) superimposed onto an RBD-hACE2 complex structure (PDB 6M0J) (97) show that the K288.2/K398.22 antibody would clash with the hACE2 receptor (indicated with a red circle). (C and G) The epitopes of K288.2 (C) and K398.22 (G) are shown in purple. CDR loops that interact with the RBD are shown in blue and labeled. (D and H) Detailed molecular interactions of K288.2 (D) or K398.22 (H) CDRs H1 and H2 with SARS-CoV-2 RBD are shown. Somatically mutated residues are indicated with *. The CDRH2 of *IGHV3–73* antibodies interacts extensively with the RBD. (**I**) The *IGHV3–73* germline sequence and the sequences of K288.2 and K398.22 are shown. The *IGHV3–73* sequence is shown in black. Only CDR sequences are shown for clarity. Lengths of CDR H3 can vary. Yellow shades indicate paratope regions in antibody heavy chain defined as buried surface area (BSA > 0 Å^2^). Red letters indicate somatic mutations. Bottom numbers indicate Kabat numbering. Y58 is mutated to E/H. Our structures show germline Y58 would clash, whereas E/H is able to interact with the RBD. (**J**) The Fab-RBD complex structures of the two antibodies are shown superimposed on the RBD. (**K**) Sequence conservation of the epitope residues in SARS-related strains recognized by *IGHV3–73*–encoded paratopes is shown (cutoff = 4 Å). SARS-CoV-2, SARS-CoV-1, WIV-1, RaTG13, and pang17 were used for the conservation analysis.

To investigate the binding of the potent macaque *IGHV3–73*–encoded nAbs in greater detail, we determined crystal structures of K288.2 and K398.22 Ab Fabs with SARS-CoV-2 RBD at 2.3 and 1.95 Å resolution, respectively (Fig. 6, A to H, and tables S1 and S2). Both nAbs bind a distinct epitope where the heavy chain dominates the interaction, as expected from enrichment of *IGVH3–73* and its pairing with different light chains (Fig. 6, A to H, and figs. S10, S14, and S15). However, the *IGHV3–73*–encoded macaque nAbs and *IGHV3–53* human nAbs interact differently with the RBD; both interact with the ACE2 binding site but at opposite ends (Fig. 6, B and F, and fig. S14, B and C). For the human nAbs, the germline-encoded CDRH1 and CDRH2 both contribute substantially to RBD recognition (18, 22); the macaque nAbs predominantly rely on CDRH2 and less on CDRH1 (Fig. 6, D, H, and I, and fig. S14, A and C to E). Furthermore, these CDRs bind to different regions on the RBD (fig. S14C). Most of the CDRH2-mediated epitope recognition by the macaque bnAbs derives from germline residues that interact with a conserved sarbecovirus RBD region (Fig. 6, I to K, and figs. S14A and S16). The human and macaque germlines also differ at critical CDRH1 residues. The human *IGHV3–53* germ line encodes a CDRH1 ^32^NY^33^ motif critical for RBD recognition (fig. S14, C and D) (22), whereas macaque *IGHV3–73* Abs bind to a different RBD region, where only V_H_ E33 in CDRH1 of K288.2 and K398.22 interact (Fig. 6, D and H, and fig. S14, C and E). Thus, immunodominant *IGHV3–73*–encoded macaque nAbs target more highly conserved RBD residues across ACE2-using sarbecoviruses, thereby providing a structural basis for their broad cross-reactivity (Fig. 6K and fig. S16).

## DISCUSSION

The emerging SARS-CoV-2 VOCs and the possibility of further SARS pandemics have increasingly focused attention on vaccines and immunization strategies to induce potent cross-bnAb responses. Natural infection and current vaccines (two immunizations, comparable to the macaque schedule) for SARS-CoV-2 in humans typically induce limited cross-neutralizing responses. The NHP model offers the possibility to explore methods to induce bnAbs because preclinical approaches to vaccine development typically rely heavily on immunization using this model (5, 45, 49–57). Here, we immunized macaques with S-protein using a strong saponin-based adjuvant and generated potent and broad nAb responses. Unlike many human studies, serum nAb responses were equally potent, for example, against SARS-CoV-2 and SARS-CoV-1, showed potent neutralization of a range of sarbecoviruses, and effectively neutralized SARS-2 VOCs, including Omicron. Some degree of breadth was also observed in S-protein-mRNA–immunized macaques and in mice immunized with the same S-protein protocol as we used in macaques.

Some of the differences observed here between macaques and humans may lie in the different naïve repertoire of the two species. Whereas the immunodominant human nAb RBS-A/class 1 response to S-protein largely uses the *IGHV3–53* germline gene segment, the immunodominant macaque response leads to a strong *IGHV3–73* response that involves a different epitope and different approach angle to interact with the RBD. The different binding mode for macaque antibodies allows for SARS-CoV-1 and SARS-CoV-2 cross-neutralization and retention of neutralization of VOCs. Other factors may contribute to the differences seen between the responses that we observe in macaques and in many human vaccine studies as discussed below.

A number of lessons can be learned from this study. First, immunization of macaques can, with a single prime and boost, lead to SARS-CoV-1 and SARS-CoV-2 cross-neutralizing responses. Therefore, the observation of broad responses to an immunogen in macaques needs careful examination, particularly for the use of the *IGHV3–73* gene. Differences between human and rhesus macaque Ab repertoires could have important consequences for vaccine model studies, and this observation has been made beyond SARS-CoV-2. For example, for HIV, VRC01-class bnAbs are encoded by human *IGHV1–2*, and no similar germlines are present in the macaque repertoire (58–60). An immunogen (eOD-GT8) specifically designed to activate human *IGHV1–2* germline–encoded naïve B cell precursors would then not be expected to produce a similar response in macaques. Second, the isolation of potent macaque cross-neutralizing Abs furnished evidence of an epitope in SARS-CoV-2 that could be targeted by immunization of humans if the immunodominance of *IGHV3–53* and other preferred germ lines can be reduced by rational immunogen engineering (61–64). Although this particular epitope has not yet been targeted by human SARS-CoV-2 Abs with known structures as far as we are aware, the human SARS-CoV-2 nAb DH1047 (65) heavy chain binds in a similar location but in a completely different orientation so that the light chains are oppositely positioned. Likewise, another previously reported Ab that broadly neutralizes various sarbecoviruses including SARS-CoV-2, S2X259 (66), also targets a similar region but with a slightly different angle of approach to the RBD than DH1047. The epitope of another nAb S2A4 (66, 67) is slightly further down the RBD. Third, we used a strong saponin-based adjuvant that may have contributed to elicitation of bnAbs, and adjuvants should be evaluated in combination with immunogens designed to induce pan-CoV nAbs, perhaps in small-scale experimental medicine studies, as emphasized elsewhere (49). Fourth, given the important response differences between human and the closely phylogenetically related rhesus macaque, continued exploration of human Ab repertoire–based model systems such as humanized mice (68–70) and specific Ab knock-in mice (63, 71, 72) could contribute to immunogen evaluation in rational vaccine design approaches.

Our study has some limitations. We used a strong saponin-based adjuvant that we have used in HIV NHP studies but has not been yet deployed in humans. Thus, a direct comparison between humans and NHPs using the same protein and adjuvant combination cannot be made. The interval that we used between prime and boost (10 weeks) was greater than used in typical two-dose human immunization studies (3 to 4 weeks in many cases) and may have contributed to the development of responses. Also, three-dose immunizations are now widely used, increasing nAb responses and the breadth of such responses (73, 74). Last, the mAbs that we describe were isolated from memory B cells. A greater breadth of nAb reactivity in memory B cell responses has been described as compared to serum nAb responses (75). The notable difference between NHPs and humans in terms of serum nAb breadth is nevertheless clear, but the possibility of some discordance between details of mAb specificities and those of the corresponding serum Abs should be borne in mind. In summary, we have shown that bnAbs can be readily induced in NHPs with S-protein immunization, and our findings provide valuable information that may ultimately assist in pan-CoV vaccine design.

## MATERIALS AND METHODS

### Study design

The objective of the study was to evaluate SMNP-adjuvanted SARS-CoV-2 S-protein–induced Ab responses in rhesus macaques and how those immune responses compare with SARS-CoV-2 infection or S-mRNA–induced Ab responses in human donors. We did not use any statistical methods to predetermine sample sizes for the animal studies. The rhesus macaque experiment used four animals per group, and the mouse experiment used five animals per group. Rhesus macaque groups were evenly distributed by sex. For the mouse study, age-matched (8-week-old) three females and two males were used. All immunological and virological measurements were performed blinded. The serum Ab titers of the SARS-2 S-protein–immunized animals were determined biweekly. No datapoints were excluded as outliers in any experiment. For in vitro binding and neutralization studies, the serum samples and mAbs were tested in duplicate, and experiments were repeated independently for rigor and reproducibility.

### Convalescent COVID-19 and SARS-CoV-2 S-protein–vaccinated human serum

Serum from convalescent COVID-19 donors (76) and from S-mRNA–vaccinated humans was provided through the “Collection of Biospecimens from Persons Under Investigation for 2019-Novel Coronavirus Infection to Understand Viral Shedding and Immune Response Study” University of California, San Diego (UCSD) Institutional Review Board (IRB) no. 200236. Protocol was approved by the UCSD Human Research Protection Program. Convalescent serum samples were collected on the basis of COVID-19 diagnosis regardless of gender, race, ethnicity, disease severity, or other medical conditions. All human donors were assessed for medical decision-making capacity using a standardized, approved assessment and voluntarily gave informed consent before being enrolled in the study. Samples from S-protein vaccine (NVX-CoV2373)–immunized individuals were collected in the San Diego region by the La Jolla Institute for Immunology. Work at the La Jolla Institute for Immunology was approved by the IRB of the La Jolla Institute for Immunology (IRB no.VD-214).

### Ethics statement

The mice and rhesus macaque animal studies were approved and carried out in accordance with protocols provided to the Institutional Animal Care and Use Committee (IACUC), respectively, at The Scripps Research Institute under approval number 19–0020 and at Alphagenesis IACUC under approval number AUP 19–10. The animals at both facilities were kept, immunized, and bled in compliance with the Animal Welfare Act and other federal statutes and regulations relating to animals and in adherence to the Guide for the Care and Use of Laboratory Animals (National Research Council, 1996).

### SMNP adjuvant preparation

SMNP adjuvant was prepared as described (77). Cholesterol (20 mg/ml; Avanti Polar Lipids, catalog no. 700000) and DPPC (1,2-dipalmitoyl-sn-glycero-3-phsophocholine: 20 mg/ml; Avanti Polar Lipids, catalog no. 850355) were dissolved in Milli-Q water containing 20% w/v MEGA-10 (Sigma-Aldrich, D6277) detergent at 60°C. Monophosphoryl lipid A (10 mg/ml; Avanti Polar Lipids, catalog no. 699800P) was dissolved in 20% w/v MEGA-10 at 37°C. Quil-A saponin (InvivoGen; vac-quil) was dissolved in Milli-Q water at a final concentration of 100 mg/ml at 37°C. All components were then mixed at a mass ratio of 10:10:2.5:1 [Quil-A:cholesterol:DPPC:MPLA (Monophosphoryl lipid A)] at 60°C followed by dilution with phosphate-buffered saline (PBS) to a final concentration of cholesterol (1 mg/ml). The solution was allowed to equilibrate over night at room temperature, followed by dialysis against PBS using a 10k molecular weight cutoff (MWCO) membrane. The adjuvant solution was then sterile filtered, concentrated using 50k MWCO centricon spin filters, and further purified by fast protein liquid chromatography using a Sephacryl S-500 HR size exclusion column. Cited concentrations of SMNP refer to the concentration of saponin injected.

### Immunization and sampling

Eight outbred, healthy Indian-origin rhesus macaques (*Macaca mulatta*) were housed at Alphagenesis. Two groups of four rhesus macaques, evenly distributed by gender between the ages 3 and 5 years, were immunized with SARS-CoV-2 S-protein immunogen as bolus prime (group 1) or ED prime (group 2) and bolus immunization boost for both groups. Animals were primed (week 0) and boosted (week 10) by subcutaneously injecting 100 μg of SARS-CoV-2 S-protein immunogen along with 375 μg of SMNP (77) per animal per immunization. All immunizations were administered subcutaneously divided between the left and right midthighs. For ED priming, animals were given seven injections of SARS-CoV-2 S-protein and the SMNP adjuvant in each thigh over 12 days (on days 0, 2, 4, 6, 8, 10, and 12). The total doses of SARS-CoV-2 S-protein immunogen at each injection during the ED prime immunization were 0.2, 0.43, 1.16, 3.15, 8.56, 23.3, and 63.2 μg distributed evenly between two immunization sites. EDTA blood was collected at various time points into CPT tubes for PBMC and plasma isolation. Serum was isolated using serum collection tubes and frozen. One group of five mice [C57BL/6 mice (The Jackson Laboratory): three females and two males], aged ~8 weeks, were immunized with 20 μg of SARS-CoV-2 S-protein immunogen along with 5 μg of SMNP adjuvant per animal per immunization. Animals were immunized twice subcutaneously by infection immunogen-adjuvant vaccine formulation at prime (week 0) and boost (week 3), and the serum samples were collected at various time points for the analysis of Ab responses. The SARS-CoV-2 S-mRNA–vaccinated NHP serum samples for comparisons were obtained from a previously published study (51).

### Plasmid construction

To generate the expression plasmids for soluble S ectodomain proteins from SARS-CoV-1 (residues 1 to 1190; GenBank: AAP13567) and SARS-CoV-2 (residues 1 to 1208; GenBank: MN908947), S-protein encoding genes were synthesized by GeneArt (Life Technologies). The ectodomains of SARS-CoV-1 and SARS-CoV-2 were constructed by polymerase chain reaction (PCR) amplification and Gibson assembly [New England Biolabs (NEB), E2621L] cloning into the vector phCMV3 (Genlantis). To stabilize soluble S-proteins in the trimeric prefusion state, we made the following changes: double proline substitutions (2P) in the S2 subunit, replacement of the furin cleavage site by “GSAS” (Glycine-Serine-Alanine-Serine)-linker in SARS-CoV-2 (residues 682 to 685) and SARS-CoV-1 (residues 664 to 667), and incorporation of a C-terminal T4 fibritin trimerization motif (78, 79). To aid purification and biotinylation, the HRV-3C protease cleavage site, 6x HisTag, and AviTag spaced by GS (Glycine-Glycine-Serine-Glycine-Glycine-Serine) linkers were added to the C terminus. To generate gene fragments encoding SARS-CoV-1 RBD (residues 307 to 513), SARS-CoV-2 NTD (residues 1 to 290), RBD (residues 320 to 527), RBD-SD1 (residues 320 to 591), and RBD-SD1–2 (residues 320 to 681) subdomains, PCR amplifications were carried out from the SARS-CoV-1 and SARS-CoV-2 plasmids. The original secretion signal or the Tissue Plasminogen Activator leader sequence was cloned in frame with the gene fragments.

### Transient transfection

To express Abs, the corresponding heavy chain and light chain plasmids were transiently transfected into the Expi293 cell (Life Technologies) at 3 × 10^6^ cells/ml with FectoPRO PolyPlus transfection reagent (Polyplus, catalog no. 116–040). To boost the protein yield, we fed cells with 300 mM sodium valproic acid solution and glucose solution 1 day after transfection. To purify mAbs, the supernatants were harvested 4 days after transfection. To express the soluble S ectodomain proteins from SARS-CoV-1, SARS-CoV-2, and their truncations, plasmids were transfected into human embryonic kidney (HEK) 293F cells (Life Technologies) at 1 million cells/ml. For 1 liter of HEK293F transfection, we combined 350 μg of plasmids with 16 ml of transfectagro (Corning) and filtered with 0.22-μm Steriflip Sterile Disposable Vacuum Filter Units (Millipore Sigma). The 1.8 ml of 40K polyethylenimine (PEI) (1mg/ml) was mixed into 16 ml of transfectagro in another conical tube. The filtered plasmid solution was gently combined with the mixed PEI solution by inverting the tube several times. After resting at room temperature for 30 min, the mixture was poured into 1 liter of HEK293F cells. The supernatant was centrifuged and filtered with 0.22-μm membrane to a glass bottle 5 days after transfection.

### Protein purification

To purify the mAbs, the Expi293 supernatant was loaded on to Protein A Sepharose (GE Healthcare) for 2 hours at room temperature or 4°C overnight. After incubation, the Protein A Sepharose was loaded into Econo-Pac columns (Bio-Rad, #7321010). One column volume of PBS was used to wash away nonspecific protein. To elute the Ab, 0.2 M citric acid (pH 2.67) was briefly mixed with Protein A Sepharose and then dripped into 2 M tris base to neutralize the protein solution. The Ab solution was buffer-exchanged into PBS using 30K Amicon tubes (Millipore, UFC903008). To purify the His-tagged proteins, the HEK293F supernatant was loaded onto HisPur Ni-NTA (nitrilotriacetic acid)-resin (Thermo Fisher Scientific). Three bed volumes of wash buffer [25 mM imidazole (pH 7.4)] were used to wash away the nonspecific binding proteins before the elution step. To elute the purified proteins from the column, 25 ml of elution buffer [250 mM imidazole (pH 7.4)] was loaded to the column at slow gravity speed (about 4 s per drop). The purified proteins were buffer-exchanged into PBS using Amicon tubes. For further purification, the proteins were subjected to size exclusion chromatography using Superdex 200 (GE Healthcare). Endotoxin from the SARS-CoV-2 S-protein immunogen was removed using Pierce High-Capacity Endotoxin Removal Resin (#88270) following the manufacturer’s directions. Briefly, 50 μl of beads was washed twice and resuspended in 1 ml of filtered PBS. Five hundred microliters of protein (2 mg/ml) was added to the resin and incubated at 4°C overnight on a rocking incubator. Protein and beads were passed through a 0.22-μm spin filter. Endosafe nexgen-MCS (Charles River) and Endosafe LAL cartridges (Charles River, #PTS201F) were used to measure endotoxin concentrations. An endotoxin concentration below 10 EU (Endotoxin Units)/ml was used for the immunizations.

### Cell lines

To generate HeLa-hACE2 cells, a human ACE2 lentivirus was used to transduce HeLa cells. To produce the ACE2 lentivirus, the pBOB-hACE2 plasmid was cotransfected into HEK293T cells with lentiviral packaging plasmids pMDL, pREV, and pVSV-G (Addgene #12251, #12253, and #8454) by Lipofectamine 2000 (Thermo Fisher Scientific, 11668019). The supernatants that contain lentivirus were collected 48 hours after transfection. The stable cell line after transduction was selected and scaled up for use in the neutralization assay. The HEK293F cell is a human embryonic kidney suspension cell line (Life Technologies). We used 293FreeStyle expression medium (Life Technologies) to culture the cell line in the shaker at 150 rpm, 37°C with 8% CO_2_. Expi293F cells are derived from the HEK293F cell line (Life Technologies). We used Expi293 Expression Medium (Life Technologies) in the same condition as the HEK293F cell line. The adherent cells were cultured in flasks or dishes in the incubator at 37°C with 8% CO_2_ with Dulbecco’s modified Eagle’s medium (Corning, catalog no. 15–013-CV) supplemented with 10% fetal bovine serum (FBS; Omega, catalog no. FB-02) and 1% penicillin-streptomycin (Corning, catalog no. 30–002-CI).

### Enzyme-linked immunosorbent assay

The 96-well half-area plates (Corning, catalog no. 3690, Thermo Fisher Scientific) were coated overnight at 4°C with mouse anti–His-tag Ab (2 μg/ml; Invitrogen, catalog no. MA1–21315-1MG; Thermo Fisher Scientific) in PBS. Plates were washed three times with PBS plus 0.05% Tween 20 (PBST) and blocked with 3% (w/v) bovine serum albumin in PBS for 1 hour. After removal of the blocking buffer, the plates were incubated with His-tagged S-proteins at a concentration of 5 μg/ml in 1% bovine serum albumin plus PBST for 1.5 hours at room temperature. After a washing step, NHP serum samples were added in threefold serial dilutions in 1% bovine serum albumin/PBST starting from 1:30 dilution and incubated for 1.5 hours. For positive controls, CR3022 was used for SARS-CoV-1 S-protein, CC12.1 for SARS-CoV-2 S-protein, and Lotus 6C serum for MERS-CoV S-protein. DEN3 was used as a negative control for all three S-proteins. Control Abs were added in threefold serial dilutions in 1% bovine serum albumin/PBST starting at 10 μg/ml. After washes, a secondary Ab conjugated with alkaline phosphatase (AffiniPure goat anti-human IgG Fc fragment specific, Jackson ImmunoResearch Laboratories, catalog no. 109–055-008) diluted 1:1000 in 1% bovine serum albumin/PBST was added to each well. After 1 hour of incubation, the plates were washed and developed using alkaline phosphatase substrate pNPP (p-Nitrophenyl phosphate) tablets (Sigma-Aldrich, catalog no. S0942–200TAB) dissolved in a stain buffer. The absorbance was measured after 10 and 20 min and was recorded at an optical density of 405 nm using a VersaMax microplate reader (Molecular Devices), where data were collected using SoftMax version 5.4 (Molecular Devices). The wells without the addition of serum served as a background control.

### Pseudovirus production

To generate pseudoviruses, the MLV-gag/pol and MLV-CMV-Luciferase plasmids were cotransfected with full-length or variant SARS-CoV-1 or SARS-CoV-2 plasmid into HEK293T cells using Lipofectamine 2000 (Thermo Fisher Scientific, 11668019). The supernatants containing pseudovirus were collected 48 hours after transfection and filtered by a 0.22-μm membrane. The pseudoviruses were stored at −80°C before use.

### Pseudovirus entry and serum neutralization assays

To test the inhibition of pseudovirus infection by serum or mAbs, we used the stable cell line HeLa-hACE2 generated by lentivirus transduction with consistent ACE2 expression to carry out the assay. To calculate the neutralization efficiency, the serum samples or mAbs were threefold serially diluted. Twenty-five microliters of each dilution was incubated with 25 μl of pseudovirus at 37°C for 1 hour in 96-well half-area plates (Corning, 3688). During the incubation time, HeLa-hACE2 cells were suspended at a concentration of 2 × 10^5^/ml with the culture medium adding diethylaminoethyl–dextran (20 μg/ml; Sigma-Aldrich, #93556–1G). After a 1-hour incubation, 50 μl of cell solution was distributed into each well (10,000 cells per well). To evaluate the neutralization efficiency, the supernatants were removed from the well 48 hours after infection. The HeLa-hACE2 cells were lysed by luciferase lysis buffer [25 mM Gly-Gly (pH 7.8), 15 mM MgSO_4_, 4 mM EGTA, and 1% Triton X-100] at room temperature for 10 to 20 min. Luciferase activity of the cells in each well was inspected after adding Bright-Glo (Promega, PRE2620) by a luminometer. All of the serum samples and mAbs were tested in duplicate for each experiment. All experiments were repeated independently at least twice. Percentage of neutralization was calculated according to the equation

$$\text{\% Neutralization }=100^{*}\left(1-\frac{\left(\text{ RLU of sample })-(\text{ Average RLU of CC }\right)}{\left(\text{ Average RLU of VC })-(\text{ Average RLU of CC }\right)}\right)$$

where RLU indicates relative light units, CC indicates cell control, and VC indicates virus control. The 50% pseudovirus neutralizing (IC_50_) or binding (ID_50_) Ab titer was calculated by nonlinear fitting the plots of luciferase signals against Ab concentrations or serum dilution ratio in GraphPad Prism.

For neutralization of anti-RBD Ab depleted macaque serum, the immune serum samples were depleted by two rounds of sequential incubation with SARS-CoV-2 RBD–conjugated with magnetic beads. Beads were conjugated to the RBD by mixing every 5 mg of streptavidin p-Nitrophenyl phosphate (SA) T1 beads (Invitrogen Dynabeads MyOne SA-T1, catalog no. 65601) with 75 μg of randomly biotinylated SARS-CoV-2 RBD protein. Before conjugation, 5 mg of SA-T1 beads was washed with 1 ml of 0.1% bovine serum albumin in 1× PBS four times and once with 1 ml of 1× PBS. A DynaMag-2 magnet was used to capture the beads during washes. For conjugation, 500 μl (500 μg/ml) of randomly biotinylated SARS-CoV-2 RBD solution was mixed with 5 mg of cleaned T1 beads. The mixture was placed on a rotator at room temperature for 1 hour. To wash away unconjugated SARS-CoV-2 RBD protein, the sample was washed three times with 1× PBS. To deplete the RBD-binding Abs from the immune serum samples, 60 μl of each serum sample was diluted fivefold and mixed with 5 mg of the RBD-conjugated magnetic beads and incubated overnight at 4°C. Another round of depletion was done using 5 mg of the RBD-conjugated magnetic beads at 4°C for 3 hours to further eliminate any remaining RBD-binding Abs. The depletion of RBD-directed Abs in the immune serum was confirmed by BLI binding to SARS-CoV-2 RBD and the S-protein.

### Bio-layer interferometry

Binding of NHP mAbs to SARS-CoV-1 and SARS-CoV-2 S-proteins, as well as truncated proteins, was analyzed on an Octet RED384 system using a Protein A biosensor (18–5010, Sartorius) to capture the mAbs. Ten micrograms per milliliter of each NHP mAbs was loaded on the hydrated biosensor for 60 s. Biosensors were subsequently moved into blank buffer (1× PBS + 0.1% Tween 20) for 60 s to remove unbound protein and provide a baseline. The biosensors were moved to immerse in the protein solutions with 200 nM S-protein or truncated proteins for 120 s to acquire an association signal. To monitor disassociation, the biosensors were moved into blank buffer for 240 s. All proteins and mAbs were diluted with 1× PBS + 0.1% Tween 20.

### In-tandem epitope binning by BLI

In-tandem epitope binning was carried out using the Octet RED384 to distinguish binding epitopes of mAbs from each other or from human mAbs or from hACE2-Fc. His-tagged SARS-CoV-2 RBD protein antigens (100 nM) were captured using Anti-HIS (HIS2) biosensors (18–5114, Sartorius). The biosensor was loaded with antigen for 5 min and then moved into the mAbs at saturating concentration of 100 μg/ml for 10 min. The biosensors were then moved into competing mAbs at a concentration of 25 μg/ml for 5 min to measure binding in the presence of saturating Abs. All reactions were done in 1× PBS + 0.1% Tween 20.

### Competition BLI

To inspect the binding epitope of the NHP serum samples compared with known human SARS-CoV-2 mAbs or hACE2-Fc, we did in-tandem epitope binning experiments using the Octet RED384 system. Randomly biotinylated SARS-CoV-2 S or RBD protein antigens (200 nM) were captured using SA-biosensors (18–5019, Sartorius). The biosensor was loaded with antigen for 5 min and then moved into the saturating mAbs at a concentration of 100 μg/ml for 10 min. The biosensors were then moved into 1:50 diluted NHP serum for 5 min to measure binding in the presence of saturating Abs. As control, biosensors loaded with antigen were directly moved into 1:50 diluted NHP serum. The percent (%) inhibition in binding is calculated with the formula: percent (%) binding inhibition = 1 − (serum binding response in the presence of the competitor Ab/binding response of the corresponding control serum Abs without the competitor Ab).

### Random biotinylation of proteins

The S-proteins and truncated proteins were randomly biotinylated using an EZ-Link *N*-hydroxysuccinimide–polyethylene glycol (PEG) Solid-Phase Biotinylation Kit (Thermo Fisher Scientific, 21440). The reagents in each tube were dissolved with 10 μl of dimethyl sulfoxide into stock solution. For the working solution, 1 μl of stock solution was diluted by 170 μl of water freshly before use. The S-protein or truncated proteins were concentrated to 7 to 9 mg/ml using Amicon tubes in PBS before biotinylation. Three microliters of working solution was added into each 30 μl of protein aliquot and incubated on ice for 3 hours. The reaction ended by buffer exchanging the protein into PBS. The biotinylated proteins were evaluated by BLI.

### BirA biotinylation of proteins for B cell sorting

To generate biotinylated S-protein probes for B cell sorting, coronavirus S-proteins were constructed with an Avi-tag at the C terminus. The Avi-tagged S-proteins were concentrated to 7 to 9 mg/ml in tris-buffered saline (TBS) before the biotinylation reaction using the BirA Biotin-Protein Ligase Reaction Kit (Avidity) following the manufacturer’s instructions. Briefly, for each reaction, 50 μl of protein solution, 7.5 μl of BioB Mix, 7.5 μl of Biotin200, and 5 μl of BirA ligase (3 mg/ml) were added in PCR tubes and mixed thoroughly. After incubating on ice for 3 hours, the biotinylated protein was purified by size exclusion chromatography. The biotinylated proteins were evaluated by BLI using the SA biosensor.

### Isolation of mAbs

The sorting strategy of antigen-specific memory B cells was described in previous papers (80–82). The sorting process was performed in a 96-well format. To enrich for antigen-specific memory B cells, PBMCs from NHPs 14 weeks after immunization were stained with fluorescently labeled Abs and the fluorophore-conjugated S-proteins. Specifically, BirA biotinylated SARS-CoV-1 S-protein was coupled to streptavidin–Alexa Fluor (AF) 647 (Thermo Fisher Scientific, S32357). The BirA biotinylated SARS-CoV-2 S-protein was coupled to streptavidin-AF488 (Thermo Fisher Scientific, S32354) and streptavidin–brilliant violet (BV) 421 (BD Biosciences, 563259) separately. The S-proteins were freshly conjugated with the streptavidin-fluorophores at 2:1 or 4:1 molecular ratio at room temperature for 30 min before use. The frozen NHP PBMCs were thawed in 10 ml of recover medium (RPMI 1640 medium containing 50% FBS) immediately before staining. The cells were washed with fluorescence-activated cell sorting (FACS) buffer (PBS, 2% FBS, and 2 mM EDTA) and counted. For each sample, 10 million cells were resuspended in 100 μl of FACS buffer and labeled with Abs for rhesus macaque surface markers. The T cell markers CD3 [allophycocyanin (APC)–Cy7; BD Pharmingen, #557757], CD4 (APC-Cy7; BioLegend, #317418), and CD8 (APC-Cy7; BD Pharmingen, #557760); the monocytes marker CD14 (APC-H7; BD Pharmingen, #561384; clone M5E2); and IgM (phycoerythrin; BioLegend, #314508; clone MHM-88) were stained for negative selection. The B cell markers CD19 [peridinin chlorophyll protein (PerCP)–Cy5.5; BioLegend, #302230; clone HIB19], CD20 (PerCP-Cy5.5; BioLegend, #302326; clone 2H7), and IgG (BV786; BD Horizon, #564230; clone G18–145) were stained to select IgG^+^ B cells. After 15 min of staining on ice, SARS-CoV-1-S-AF647, SARS-CoV-2-S-AF488, and SARS-CoV-2-S-BV421 were added to the staining solution. After another 30 min of incubation on ice, FVS510 Live/Dead stain (Thermo Fisher Scientific, #L34966) diluted 1:1000 with FACS buffer was added into the staining solution. Fifteen minutes later, the stained cells were washed with cold 10 ml of FACS buffer and resuspended in 500 μl of FACS buffer for each 10 million cells. After filtering the cells through a 70-μm mesh cap FACS tube (Thermo Fisher Scientific, 08–771-23), the cells were sorted using a BD FACSMelody (BRV 9 Color Plate 4way). To isolate the SARS-CoV-1-S and SARS-CoV-2-S cross-reactive B cells, the gating strategy was designed as follows: After gating the lymphocytes [side-scatter area (SSC-A) versus forward scatter area (FSC-A)] and singlets [forward scatter height (FSC-H) versus FSC-A], live cells were selected by negative gating of FVS510 Live/Dead staining. The CD3-, CD4-, CD8-, and CD14-negative and CD19- and CD20-positive cells were gated as “CD19/CD20^+^, Dump^−^.” By selecting the IgG-positive and IgM-negative cells, the cells in the “IgG^+^, IgM^−^” gate were sequentially selected for SARS-CoV-2-S-BV421/SARS-CoV-2-S-AF488 double-positive and SARS-CoV-1-S-AF647/SARS-CoV-2-S-AF488 double-positive reactivity. The selected cells were sorted into 96-well plates individually, and plates were moved onto dry ice immediately after sorting. cDNA was generated from the sorted cells on the same day to avoid degradation of RNA. The Superscript IV Reverse Transcriptase (Thermo Fisher Scientific), dNTPs (Deoxy-nucleoside triphosphate) (Thermo Fisher Scientific), random hexamers (Gene Link), Ig gene-specific primers, dithiothreitol, and RNAseOUT (Thermo Fisher Scientific) were used in the lysis buffer containing IGEPAL (Sigma-Aldrich) to carry out reverse transcription PCR as described previously (23). To amplify IgG heavy and light chain variable regions, cDNA of each single cell was used as a template in two rounds of nested PCR reactions using hot start DNA polymerases (QIAGEN and Thermo Fisher Scientific) and rhesus macaque primers as described previously (82). The PCR products were purified with SPRI (Solid Phase Reversible Immobilization)-beads according to the manufacturer’s instructions (Beckman Coulter). Purified DNA fragments of heavy and light chain variable regions were subsequently cloned into expression vectors encoding human IgG1 and Ig kappa/lambda constant domains, respectively, using Gibson assembly (NEB, E2621L) according to the manufacturer’s instructions. Paired heavy and light chains were sequenced and analyzed using the rhesus macaque (*M. mulatta*) germline database from (59). The paired plasmids were cotransfected into 293Expi cells for Ab expression.

### Fab production

To generate the Fab of an IgG, a stop codon was inserted in the heavy chain constant region at “KSCDK*.” The truncated heavy chains were cotransfected with the corresponding light chains in Expi293 cells to produce the Fab. The supernatants were harvested 4 days after transfection. Fabs were purified with CaptureSelect CH1-XL MiniChrom Columns (#5943462005). Supernatants were loaded onto columns using an Econo Gradient Pump (Bio-Rad, #7319001). After a wash with 1× PBS, Fabs were eluted with 25 ml of 50 mM acetate (pH 4.0) and neutralized with 2 M tris base. The eluate was buffer-exchanged with 1× PBS in 10K Amicon tubes (Millipore, UFC901008) and filtered with a 0.22-μm spin filter.

### Negative-stain electron microscopy

S-protein was complexed with Fab at three times molar excess per trimer and incubated at room temperature for 30 min. Complexes were diluted to 0.02 mg/ml in 1× TBS, and 3 μl was applied to a 400-mesh Cu grid, blotted with filter paper, and stained with 2% uranyl formate. Micrographs were collected on a Thermo Fisher Scientific Tecnai Spirit microscope operating at 120 kV with a FEI Eagle charge-coupled device (4k) camera at 52,000 magnification using Leginon automated image collection software (83). Particles were picked using DogPicker (84), and three-dimensional (3D) classification was done using Relion 3.0 (85).

### Crystal structure determination of Fab-RBD complexes

The coding sequence for RBD (residues 333 to 529) of the SARS-CoV-2 S-protein was synthesized and cloned into a customized pFastBac vector (86), which was designed to fuse an N-terminal gp67 signal peptide and C-terminal His_6_-tag to the target protein. To express the RBD protein, a recombinant bacmid DNA was generated from the sequencing-confirmed pFastBac construct using the Bac-to-Bac system (Life Technologies). Baculovirus was generated by transfecting purified bacmid DNA into Sf9 cells using FuGENE HD (Promega) and subsequently used to infect suspension cultures of High Five cells (Life Technologies) at a multiplicity of infection of 5 to 10. Infected High Five cells were incubated at 28°C with shaking at 110 rpm for 72 hours for protein expression. RBD protein that was secreted into the supernatant was then concentrated using a 10-kDa MWCO Centramate cassette (Pall Corporation). The RBD protein in the concentrate was purified by affinity chromatography using Ni-NTA resin (QIAGEN), followed by size exclusion chromatography on a HiLoad Superdex 200 pg column (GE Healthcare), and buffer-exchanged into 20 mM tris-HCl (pH 7.4) and 150 mM NaCl using the same protocol as before (87). Crystallization trials were set up for SARS-CoV-2 RBD in complex with K288.2 and K398.22 Fabs. The Fab-RBD complexes were formed by mixing the two components in an equimolar ratio and incubating overnight at 4°C before setting up crystal trials. The complexes (13 mg/ml) were screened for crystallization using the 384 conditions of the JCSG Core Suite (QIAGEN) on our high-throughput robotic CrystalMation system (Rigaku) at The Scripps Research Institute by the vapor diffusion method in sitting drops containing 0.1 μl of protein and 0.1 μl of reservoir solution. For the K288.2-RBD complex, optimized crystals were then grown in 0.1 M sodium cacodylate (pH 6.5), 40% (v/v) 2-methyl-2,4-pentanediol, and 5% (w/v) PEG 8000 at 20°C. Crystals were grown for 7 days and then flash cooled in liquid nitrogen. Diffraction data were collected at cryogenic temperature (100 K) at the Stanford Synchrotron Radiation Lightsource (SSRL) on the Scripps/Stanford beamline 12–1 with a wavelength of 0.9793 Å and processed with HKL2000 (88). For the K398.22-RBD complex, optimized crystals were then grown in 0.2 M ammonium formate and 20% (w/v) PEG 3350 at 20°C. Crystals were grown for 7 days, preequilibrated with 10% (v/v) ethylene glycol, and then flash-cooled in liquid nitrogen. Diffraction data were collected at cryogenic temperature (100 K) at beamline 23ID-D of the Advanced Photon Source at the Argonne National Laboratory with a wavelength of 0.9793 Å and processed with XDS (89). Structures were solved by molecular replacement using PHASER (90) with Protein Data Bank (PDB) 7LOP for the RBD (28), whereas the models for the Fabs were generated by Repertoire Builder (https://sysimm.ifrec.osaka367u.ac.jp/rep_builder/) (91). Iterative model building and refinement were carried out in COOT (92, 93), respectively.

### Phylogenetic analysis

Heavy chain sequences of neutralizing and non-nAbs collected from animals K398 and K288 were processed using DiversityAnalyzer tool (94). *IGHV*, *IGHD*, and *IGHJ* genes of rhesus monkey (*M. mulatta*) described in (59) were used as the database of germline immunoglobulin genes. For each heavy chain sequence, the number of SHMs was computed as the number of differences in the V segment with respect to the closest *IGHV* gene from the database. Phylogenetic trees derived from heavy chain sequences of Abs collected from animals K398 and K288 were constructed using ClusterW2 tool (95) and visualized using Iroki tool (96).

### Statistical analysis

Raw, individual-level data are shown in data file S1. Statistical analysis was performed using GraphPad Prism 8 for Mac, GraphPad Software. ID_50_ neutralization titers between two groups were compared using the nonparametric unpaired Mann-Whitney two-tailed test. The correlation between two groups was determined by Spearman rank test. Data were considered statistically significant at *P* < 0.05.

## Supplementary Material

Supplementary Figures S1-S16 & Supplementary Tables S1-S2

MDAR reproducibility checklist

excel_data_file_s1

zip_data_file_s1

## Data Availability

All data associated with this study are in the paper or the Supplementary Materials. Antibody sequences have been deposited in GenBank under accession numbers OM489482-OM489531 and OM460101-OM460130. Antibody plasmids are available from R.A. or D.R.B. under a standard material transfer agreement with The Scripps Research Institute. The x-ray coordinates and structures of the sarbecovirus antibody Fabs, K288.2 and K398.22, in complex with SARS-CoV-2 RBD have been deposited to the RCSB Protein Data Bank and are available under the accession codes PDB ID 7TP3 and 7TP4, respectively. The EM maps of RBD bnAb Fabs in complex with SARS-CoV-1 and SARS-CoV-2 S-proteins have been deposited in the Electron Microscopy Data Bank (EMDB) under accession codes EMD-26522 to EMD-26539.
