# Supplementary material for: Broadly neutralizing antibodies to SARS-related viruses can be readily induced in rhesus macaques
Source: Sci Transl Med. Author manuscript; Available in PMC 2023 Aug 10. (PMC10069796; doi:10.1126/scitranslmed.abl9605)
Supplement: Supplementary Figures S1-S16 & Supplementary Tables S1-S2 [file NIHMS1873750-supplement-Supplementary_Figures_S1-S16___Supplementary__Tables_S1-S2.pdf]

Science Translational Medicine
AAAS

Supplementary Materials for

Broadly neutralizing antibodies to SARS-related viruses can be readily induced in rhesus macaques

Wan-ting He *et al.*

Corresponding author: Ian A. Wilson, wilson@scripps.edu; Dennis R. Burton, burton@scripps.edu; Raiees Andrabi, andrabi@scripps.edu

Sci. Transl. Med. **14**, eabl9605 (2022)
DOI: 10.1126/scitranslmed.abl9605

The PDF file includes:

Figs. S1 to S16
Tables S1 and S2

Other Supplementary Material for this manuscript includes the following:

MDAR Reproducibility Checklist
Data file S1

Supplementary Materials

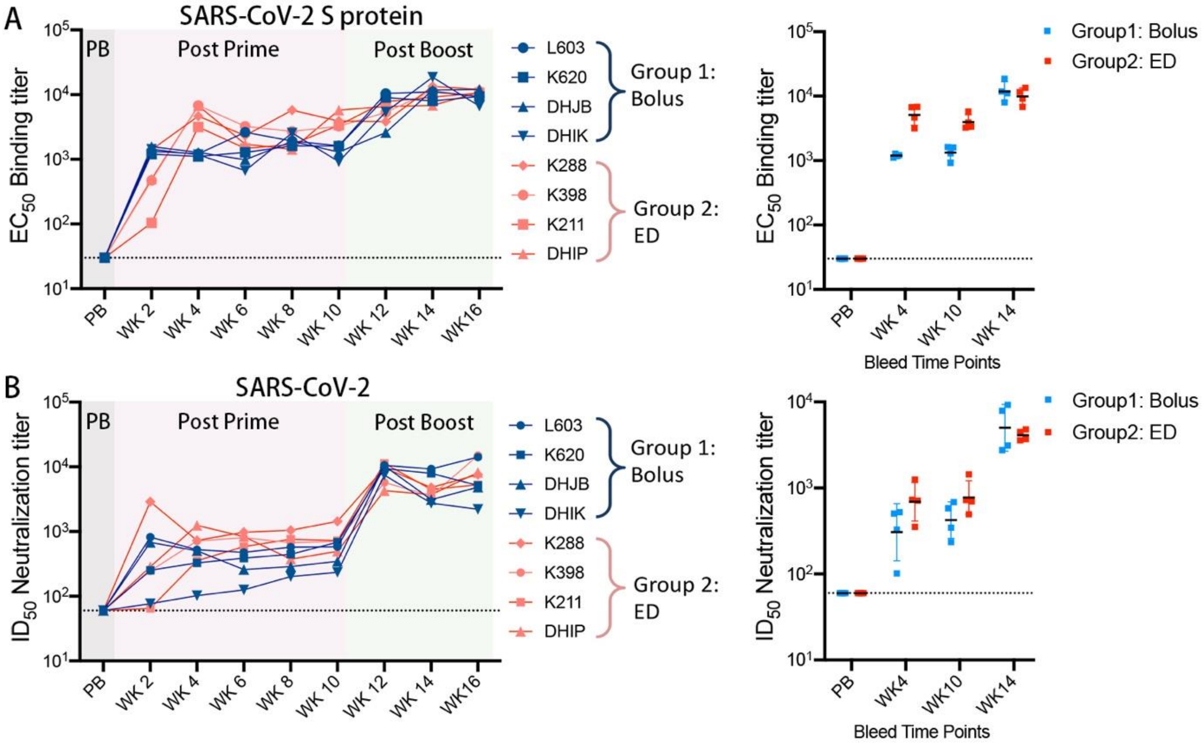

Fig. S1. SARS-CoV-2 spike (S)-protein prime-boost immunization in rhesus macaques induces strong antibody (Ab) binding and neutralizing responses.

(A) EC_{50} (half maximal effective concentration) enzyme-linked immunosorbent assay (ELISA) binding titers are shown for SARS-CoV-2 S-protein prime (WK 2, 4, 6, 8, and 10) and boosted (WK 12, 14, and 16) serum antibody responses to SARS-CoV-2 S-protein. Serum samples from animals receiving either bolus (Group 1: blue) or escalating dose (ED, Group 2: red) immunization prime (n=4 animals per group) were tested for ELISA binding. PB indicates pre-bleed serum collected pre-immunization at WK 0. Dot plots on the right show EC_{50} binding comparisons between bolus and ED groups at 4 time points: PB, post-prime (WK 4 and WK 10) and post-boost (WK 14) immunizations time points. Dashed horizontal lines at 30 indicates the lower limit of detection.

(B) SARS-CoV-2 virus-specific serum ID_{50} (50% inhibitory dose) neutralizing antibody titers were measured in SARS-CoV-2 S-protein prime (WK 2, 4, 6, 8, and 10) and boosted (WK 12, 14, and 16) animals in the bolus (Group 1: blue) and ED (Group 2: red) immunization groups. Dot plots on the right show the comparison of ID_{50} neutralization titers between bolus and ED groups at 4 time points: PB, post-prime (WK 4 and WK 10) and post-boost (WK 14) immunization time points. The dashed line at 60 indicates the lower limit of detection.

Binding: EC_{50}

SARS-CoV-2 S-protein

	L603	K620	DHJB	DHIK	K288	K398	K211	DHIP
**PB**	<100	<100	<100	<100	<100	<100	<100	<100
**WK 2**	1328	1202	1571	1428	1405	472	104	496
**WK 4**	1216	1110	1282	1179	4656	6832	3171	6743
**WK 6**	2628	1276	983	671	2329	3242	1478	1715
**WK 8**	1914	1612	1897	2561	5766	2711	1699	1425
**WK 10**	1625	1603	1293	917	3898	3249	3372	5765
**WK 12**	10397	9042	2597	5399	3836	5298	7823	6546
**WK 14**	11119	8003	11800	18643	13446	11604	9120	6818
**WK16**	9322	9391	12049	6638	12003	9349	10649	10866

SARS-CoV-1 S-protein

	L603	K620	DHJB	DHIK	K288	K398	K211	DHIP
**PB**	<100	<100	<100	<100	<100	<100	<100	<100
**WK 2**	203	215	274	314	810	183	52	197
**WK 4**	482	489	460	359	3893	4102	1381	2401
**WK 6**	968	524	410	341	2404	2746	803	859
**WK 8**	1094	719	664	969	3055	1425	776	889
**WK 10**	1034	739	593	491	2987	2428	1757	2165
**WK 12**	15055	5907	6540	7714	7843	8947	7365	5727
**WK 14**	6756	7102	7657	9430	13017	7749	5369	3303
**WK16**	4782	5410	4833	2762	9628	7297	5212	3952

Neutralization: ID_{50}

SARS-CoV-2

	L603	K620	DHJB	DHIK	K288	K398	K211	DHIP
**PB**	<60	<60	<60	<60	<60	<60	<60	<60
**WK 2**	818	252	679	77	2883	247	65	285
**WK 4**	522	328	504	102	730	712	353	1247
**WK 6**	478	387	258	126	978	806	583	836
**WK 8**	573	445	288	201	1045	671	759	373
**WK 10**	580	682	346	234	1440	701	722	494
**WK 12**	10548	9403	10391	7464	9590	5745	11283	4263
**WK 14**	9260	7913	3116	2751	4776	3579	4472	3706
**WK16**	14165	5143	4843	2203	7582	15005	5270	8200

SARS-CoV-1

	L603	K620	DHJB	DHIK	K288	K398	K211	DHIP
**PB**	<60	<60	<60	<60	85	<60	<60	<60
**WK 2**	117	243	168	155	537	80	23	97
**WK 4**	195	424	200	243	3013	1103	244	707
**WK 6**	360	529	212	275	3370	1486	352	1228
**WK 8**	400	500	202	416	2821	772	578	1003
**WK 10**	355	727	209	378	3051	775	504	605
**WK 12**	5735	11273	9691	6601	28262	15693	7289	8881
**WK 14**	3695	4437	4046	2377	12522	10843	4475	4431
**WK16**	2641	3272	3520	1246	7242	6629	1871	1922

Fig. S2. Binding and neutralization of rhesus macaque immune serum samples. SARS-CoV-2 S-protein prime-boost immunization in rhesus macaques induced strong antibody binding and neutralizing responses against SARS-CoV-2 and cross-reactive binding and neutralizing responses against SARS-CoV-1. EC_{50} values for the SARS-CoV-2 S-protein primed (WK 2, 4, 6, 8, and 10) and boosted (WK 12, 14, and 16) serum Ab responses to SARS-CoV-2 and SARS-CoV-1 S-proteins are shown on the left. SARS-CoV-2 and SARS-CoV-1 specific serum ID_{50} neutralizing antibody titers are shown on the right.

COVID-19 convalescent donors				
Sample #	SARS-CoV-2	SARS-CoV-1	Days Post-Symptom Onset	Disease Severity
CC1	49	<20	N/A	Moderate to severe
CC2	<20	<20	N/A	mod to severe
CC3	<20	<20	N/A	N/A
CC4	2320	90	N/A	Mild
CC5	<20	<20	N/A	N/A
CC6	1509	<20	13	severe
CC7	<20	<20	N/A	N/A
CC8	<20	<20	20	mild
CC9	38	<20	25	mild
CC10	196	<20	22	mild to moderate
CC11	85	<20	25	mild
CC12	630	<20	25	mild
CC13	38	<20	25	Mild
CC18	23	<20	23	mild
CC20	<20	<20	N/A	N/A
CC21	1941	<20	18	severe
CC22	406	<20	6	critical
CC23	32	<20	18	moderate
CC24	24	<20	24	mild
CC25	702	<20	27	mild
CC26	<20	<20	34	mild
CC27	51	<20	21	mild
CC28	<20	<20	30	Mild
CC29	<20	<20	30	Mild
CC30	34	<20	27	mild to moderate
CC31	<20	<20	33	Mild
CC32	<20	<20	31	Moderate
CC33	<20	<20	22	Moderate
CC34	97	<20	31	Mild
CC35	214	<20	30	Moderate to severe
CC36	115	<20	36	Moderate to severe
CC37	33	<20	64	Mild
CC38	72	<20	35	moderate
CC39	46	<20	44	Mild
CC40	151	<20	32	moderate
CC41	44	<20	31	Moderate
CC42	44	<20	21	mild
CC43	<20	<20	67	mild
CC44	<20	<20	50	Mild

S-mRNA vaccinated human donors			
Sample #	SARS-CoV-2	SARS-CoV-1	Days Post 2nd Vaccine Dose
CC102	185	<60	39
CC103	544	<60	63
CC104	263	<60	60
CC105	870	<60	48
CC106	789	<60	36
CC107	303	<60	36
CC108	486	<60	60
CC109	288	<60	60
CC110	163	<60	62
CC111	76	<60	76

S-protein vaccinated mouse			
Sample#	SARS-CoV-2	SARS-CoV-1	Days Post 2nd Vaccine Dose
#1	19498	692	14
#2	6026	166	14
#3	8128	204	14
#4	10471	398	14
#5	21379	91	14

S-protein human vaccinee serum			
Sample#	SARS-CoV-2	SARS-CoV-1	Days Post 2nd Vaccine Dose
6082-VA-3.5	100	<60	24
6082-VA-6	101.8	<60	42
6085-VA-U	<60	<60	N/A
6090-VA-6	479.6	<60	42
6094-VA-U	118	<60	N/A
6096-VA-3.5	79.13	<60	24
6097-VA-3.5	701.6	<60	24
6097-VA-6	109.2	<60	42
6099-VA-3.5	299.2	<60	24
6099-VA-6	90.55	<60	42
6259-VA-6	68.89	<60	42
6261-VA-6	<60	<60	42

S-mRNA macaque immune serum			
Sample#	SARS-CoV-2	SARS-CoV-1	Days Post 2nd Vaccine Dose
16C222	316.8	<60	28
36186	1471	173.4	28
16C235	1912	<60	28
16C237	1042	<60	28
16C283	2883	145.9	28
16C301	3862	111.7	28
16C303	1817	<60	28
34941	2708	138.6	28

Fig. S3. Neutralization of SARS-CoV-1 and SARS-CoV-2 by indicated immune serum samples. Neutralizing titers (ID_{50}) of serum antibodies are shown for COVID-19 convalescent patients (n = 39), S-mRNA vaccinated human donors (n = 10), S-protein immunized mice (n = 5), S-protein human vaccinee serum (n = 12) and S-mRNA macaque immune serum (n = 8). Neutralization was tested against SARS-CoV-2 and SARS-CoV-1.

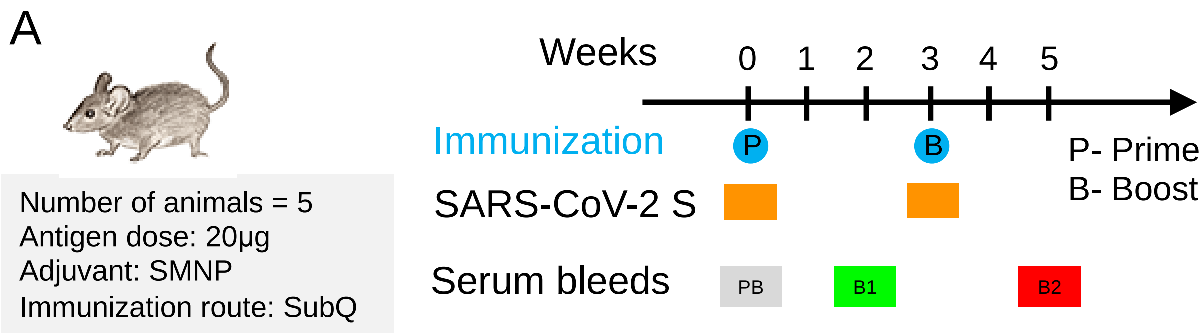

B

	M1	M2	M3	M4	M5
	SARS-CoV-2				
Pre-bleed	<50	<50	<50	<50	<50
WK 2	3318	870	1402	2011	1972
WK 5	19498	6026	8128	10471	21379
	SARS-CoV-1				
Pre-bleed	<50	<50	<50	<50	<50
WK 2	<50	<50	<50	<50	<50
WK 5	692	166	204	398	91

ID_{50} neutralization titers

Fig. S4. SARS-CoV-2 S-protein immunization in WT B6 mice and induction of neutralizing antibody responses against SARS-CoV-2 and SARS-CoV-1.
(A) Immunization of C57BL/6 mice with SARS-CoV-2 S-protein at WK 0 (P - prime) and WK 3 (B - boost). Groups of 5 mice were subcutaneously immunized twice (WK 0 prime and WK 3 boost) with 20µg of SARS-CoV-2 S-protein along with a saponin-based SMNP (Isco-MPLA (Monophosphoryl-Lipid A)) adjuvant.
(B) Neutralization of SARS-CoV-2 and SARS-CoV-1 by pre-bleed (PB), post-prime (B1, WK 2) and post-boost (B2, WK 5) immune serum was measured. Serial dilutions of serum (starting dilution of 1:50) were tested against SARS-CoV-2 and SARS-CoV-1 pseudotyped viruses in an angiotensin converting enzyme 2 (ACE2) expressing cell-based assay and ID_{50} neutralization titers are shown.

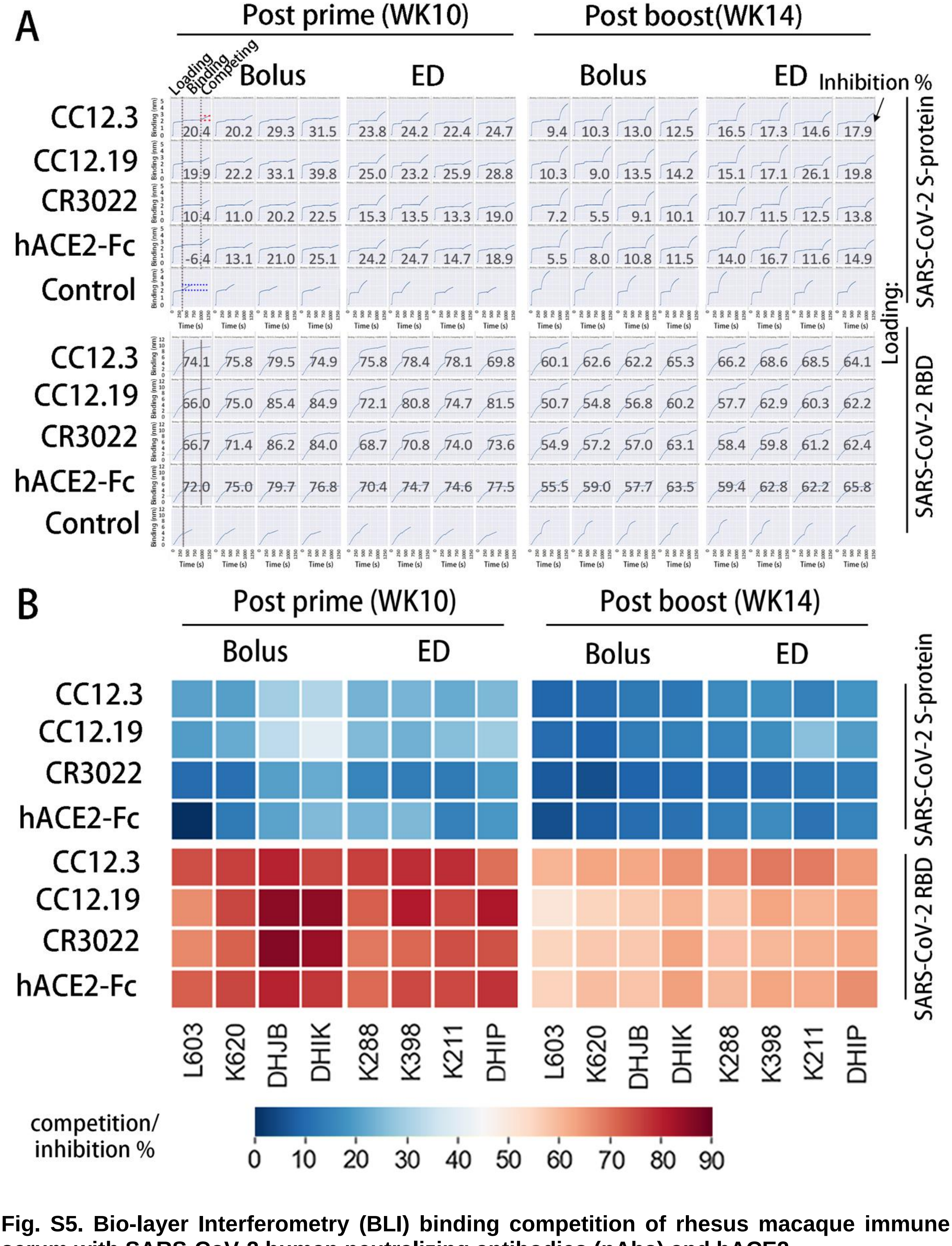

Fig. S5. Bio-layer Interferometry (BLI) binding competition of rhesus macaque immune serum with SARS-CoV-2 human neutralizing antibodies (nAbs) and hACE2.

(A) Serum from the SARS-CoV-2 S-protein immunized rhesus macaques post-prime (week 10) and post-boost (week 14) were evaluated for epitope competition with human SARS-CoV-2-specific mAbs and recombinant human hACE2 protein using BLI. Biotinylated SARS-CoV-2 S-protein (top rows) or receptor binding domain (RBD, bottom rows) molecules were captured using Streptavidin (SA) biosensors and incubated with the indicated mAbs at a saturating concentration of 100ug/mL for 10 minutes followed by an incubation for 5 minutes in 1:50 diluted serum from SARS-CoV-2 S-protein immunized rhesus macaques. As controls, the biotinylated SARS-CoV-2 S-proteins or RBDs were captured using SA biosensors and incubated only in 1:50 diluted serum from S-protein immunized rhesus macaques. BLI raw traces are shown. The percent (%) binding inhibition is calculated with the formula: [percent (%) of inhibition in the BLI response = 1- (serum binding response in presence of the competitor antibody, indicated by red dash line / response of the corresponding control serum antibodies without the competitor antibody, indicated by blue dash line)]**.**
(B) The heatmap shows BLI competition-based epitope binning of NHP immune serum with human RBD-specific nAbs CC12.3, CC12.19, and CR3022, respectively, as well as with soluble hACE2-Fc. The competition experiments were performed with full-length SARS-CoV-2 S-protein and the monomeric SARS-CoV-2 RBD (CoV-2 RBD).

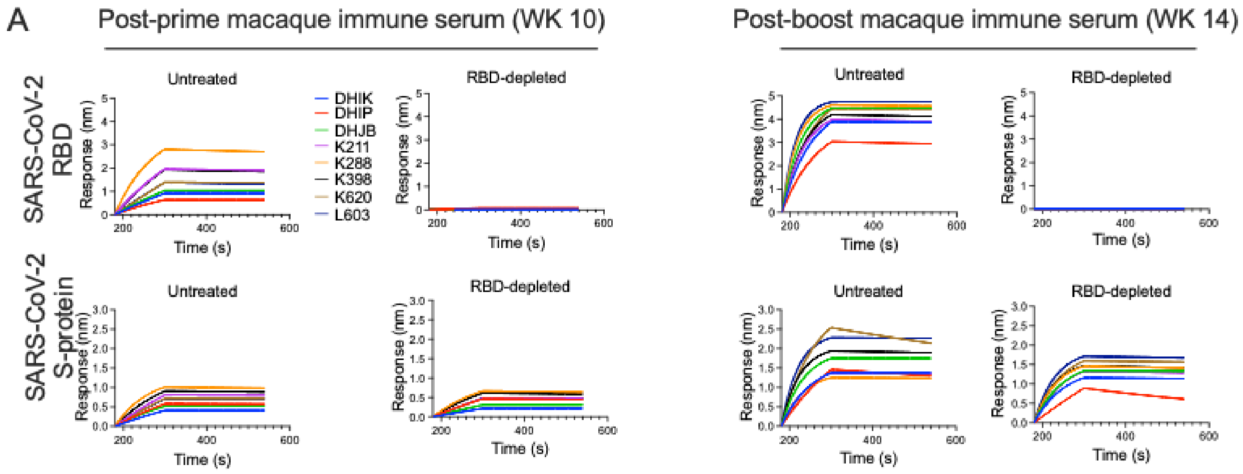

B

		Post-prime macaque immune serum (Week 10)								Post-boost macaque immune serum (Week 14)							
SARS-CoV-2 RBD	Serum	L603	K620	DHJB	DHIK	K288	K398	K211	DHIP	L603	K620	DHJB	DHIK	K288	K398	K211	DHIP
	Untreated	1.27	1.26	0.94	0.84	2.69	1.81	1.83	0.61	4.68	4.45	4.34	3.73	4.54	4.05	3.84	2.92
	RBD-depleted	0.00	0.00	0.00	0.00	0.00	0.00	0.00	0.10	0.00	0.00	0.00	0.00	0.00	0.00	0.00	0.00
	% depletion	100%	100%	100%	100%	100%	100%	100%	84%	100%	100%	100%	100%	100%	100%	100%	100%

SARS-CoV-2 S-protein	Serum	L603	K620	DHJB	DHIK	K288	K398	K211	DHIP	L603	K620	DHJB	DHIK	K288	K398	K211	DHIP
	Untreated	0.63	0.63	0.43	0.31	0.96	0.83	0.71	0.52	2.23	2.21	1.65	1.27	1.18	1.89	1.27	1.29
	RBD-depleted	0.43	0.39	0.24	0.18	0.62	0.56	0.41	0.41	1.67	1.53	1.27	1.11	1.42	1.43	1.26	0.69
	% depletion	32%	38%	44%	44%	35%	33%	42%	20%	25%	31%	23%	13%	-20%	24%	0%	47%

Fig. S6. Depletion of RBD-specific antibodies in post-prime and post-boost macaque immune serum was confirmed by BLI binding to monomeric SARS-CoV-2 RBD and SARS-CoV-2 S-protein.

(A) BLI binding curves are shown for post-prime (WK 10) and post-boost (WK 14) macaque immune serum with monomeric SARS-CoV-2 RBD and SARS-CoV-2 S-protein. Binding of both untreated and anti-RBD antibody adsorbed (RBD-depleted) immune serum samples are shown. The BLI revealed complete loss of binding to monomeric RBD in the anti-RBD depleted serum in both post-prime and post-boost immune serum.

(B) The summary table shows the BLI binding responses and percent (%) depletion of anti-RBD antibodies in post-prime and post-boost immune serum upon adsorption of polyclonal antibodies with monomeric RBD. The BLI binding responses of the untreated and anti-RBD antibody adsorbed (RBD-depleted) immune serum with monomeric SARS-CoV-2 RBD and SARS-CoV-2 S-protein are shown. The % depletion of anti-RBD antibodies for each immune serum is calculated with the formula: [Percent (%) depletion = 1- (BLI binding response of the RBD-depleted immune serum / BLI binding response of the untreated immune serum) X 100]

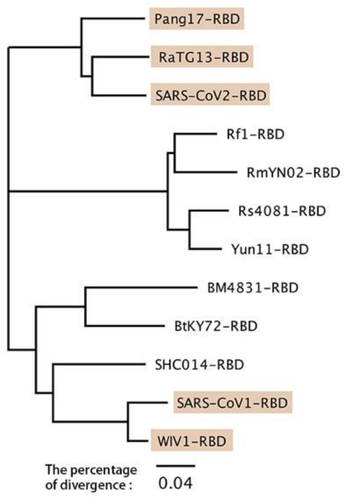

	Pang17-RBD	RaTG13-RBD	SARS-CoV2-RBD	Rf1-RBD	RmYN02-RBD	Rs4081-RBD	Yun11-RBD	BM4831-RBD	BtKY72-RBD	SHC014-RBD	SARS-CoV1-RBD	WIV1-RBD
Pang17-RBD	100.0	88.5	87.0	63.9	61.5	63.9	63.5	67.5	71.3	76.4	75.0	75.0
RaTG13-RBD	88.5	100.0	89.4	62.5	61.1	62.5	62.5	67.5	70.8	76.0	75.0	76.4
SARS-CoV2-RBD	87.0	89.4	100.0	63.5	61.5	62.5	63.0	68.9	71.8	76.0	73.1	75.5
Rf1-RBD	63.9	62.5	63.5	100.0	89.9	89.4	88.9	63.2	63.8	62.8	62.8	63.8
RmYN02-RBD	61.5	61.1	61.5	89.9	100.0	88.4	88.9	60.3	63.3	62.3	62.3	63.3
Rs4081-RBD	63.9	62.5	62.5	89.4	88.4	100.0	93.1	60.8	63.3	61.4	61.8	63.8
Yun11-RBD	63.5	62.5	63.0	88.9	88.9	93.1	100.0	61.3	64.7	62.3	62.8	64.7
BM4831-RBD	67.5	67.5	68.9	63.2	60.3	60.8	61.3	100.0	80.7	75.0	72.1	73.6
BtKY72-RBD	71.3	70.8	71.8	63.8	63.3	63.3	64.7	80.7	100.0	75.6	74.2	75.1
SHC014-RBD	76.4	76.0	76.0	62.8	62.3	61.4	62.3	75.0	75.6	100.0	80.7	83.1
SARS-CoV1-RBD	75.0	75.0	73.1	62.8	62.3	61.8	62.8	72.1	74.2	80.7	100.0	94.2
WIV1-RBD	75.0	76.4	75.5	63.8	63.3	63.8	64.7	73.6	75.1	83.1	94.2	100.0

Fig. S7. Phylogenetic relatedness of receptor binding domains of major sarbecovirus lineages.

(A) Phylogenetic tree derived from amino-acid sequences of RBD regions of S-proteins of 12 representative sarbecovirus lineages. The five taxa highlighted in beige are SARS-like viruses that utilize ACE2 receptor for infection of the host cell and thus tested in neutralization assays.

(B) Percent (%) identify matrix based on amino-acid sequences of RBD show relatedness among the sarbecovirus RBDs. Among the viruses tested for neutralization, SARS-CoV-2 RBD is closer to RatG13 (89.4%) and Pang17 (87%) RBDs compared to SARS-CoV-1 (73.1%) and WIV1 (75.5%) RBDs. WIV1 RBD is closer to SARS-CoV-1 (94.2%).

BLI response: nm

Ab_ID	SARS-CoV-2 S-protein	SARS-CoV-2 NTD	SARS-CoV-2 RBD	SARS-CoV-2 RBD-SD1	SARS-CoV-2 RBD-SD1-SD2	SARS-CoV-1 S-protein	SARS-CoV-1 RBD
K398.5	0.51	0.01	0.92	1.04	0.70	0.47	0.81
K398.22	0.32	0.03	0.71	0.91	0.52	0.33	0.94
K398.24	0.41	0.03	0.76	0.94	0.55	0.34	0.88
K398.8	0.34	0.01	0.59	0.65	0.37	0.27	0.84
K398.25	1.59	0.02	0.62	1.09	1.27	1.24	0.58
K398.10	1.17	0.01	0.80	1.00	0.87	1.12	0.53
K398.21	1.36	0.00	0.81	1.21	1.15	1.13	0.34
K398.16	1.75	-0.01	0.66	0.90	1.10	1.64	0.57
K398.23	0.80	0.03	0.93	1.27	1.11	0.40	0.94
K398.18	1.35	0.02	0.86	1.00	0.91	1.24	0.95
K398.17	1.29	0.01	0.70	0.95	1.12	1.12	0.66
K398.32	0.43	0.03	0.14	0.18	0.19	0.42	1.02
K398.9	1.62	0.72	0.02	0.02	0.03	1.04	0.01
K398.1	0.95	0.00	0.02	0.02	0.02	0.96	0.00
K398.2	1.34	-0.01	0.01	0.01	0.04	1.59	0.01
K398.3	0.96	0.00	0.02	0.02	0.02	0.96	0.00
K398.4	0.77	0.01	0.02	0.02	0.03	0.52	0.01
K398.6	1.36	-0.01	0.01	0.01	0.00	1.54	-0.01
K398.7	0.92	0.01	0.01	0.03	0.03	1.24	0.01
K398.11	1.65	0.02	0.01	0.02	0.03	1.31	0.00
K398.12	1.78	0.00	0.00	0.02	0.02	1.38	-0.01
K398.13	1.71	-0.01	0.01	0.02	0.01	1.43	0.00
K398.14	1.46	-0.02	0.00	0.00	0.01	1.72	-0.01
K398.15	1.44	-0.01	-0.01	0.00	0.01	1.72	0.01
K398.19	0.09	0.02	0.01	0.02	0.02	0.02	0.00
K398.20	0.02	0.01	-0.01	0.00	0.04	0.00	-0.02
K398.26	0.02	0.02	0.05	0.02	0.02	0.01	0.01
K398.27	1.57	0.02	0.01	0.02	0.03	1.22	0.01
K398.28	0.01	0.03	0.02	0.03	0.04	0.01	0.01
K398.29	1.40	0.02	0.03	0.02	0.03	1.65	0.01
K398.30	1.24	0.02	0.01	0.03	0.03	1.12	0.01
K398.31	0.83	0.00	0.00	0.01	0.00	0.67	0.00
K288.7	0.26	0.03	0.67	0.83	0.49	0.20	0.78
K288.8	1.41	0.03	0.76	1.28	1.26	1.50	0.74
K288.3	0.69	0.02	0.70	0.80	0.62	0.33	0.17
K288.2	0.19	0.02	0.57	0.61	0.34	0.11	0.72
K288.1	0.46	0.00	0.13	0.17	0.22	0.37	0.12
K288.4	1.47	0.01	0.02	0.02	0.02	1.07	0.01
K288.5	0.01	0.03	0.02	0.03	0.03	0.01	0.01
K288.6	0.01	0.03	0.02	0.02	0.03	0.01	0.01

Neutralization: IC_{50}

Ab_ID	SARS-CoV-1	SARS-CoV-2
K398.5	0.007	0.049
K398.22	0.008	0.077
K398.24	0.009	0.082
K398.8	0.011	0.089
K398.25	0.069	0.134
K398.10	1.788	0.165
K398.21	>5	0.222
K398.16	0.136	0.244
K398.23	1.046	0.279
K398.18	0.108	0.322
K398.17	0.191	0.415
K398.32	0.011	0.711
K398.9	>5	4.812
K398.1	>5	>5
K398.2	>5	>5
K398.3	>5	>5
K398.4	>5	>5
K398.6	>5	>5
K398.7	>5	>5
K398.11	>5	>5
K398.12	>5	>5
K398.13	>5	>5
K398.14	>5	>5
K398.15	>5	>5
K398.19	>5	>5
K398.20	>5	>5
K398.26	>5	>5
K398.27	>5	>5
K398.28	>5	>5
K398.29	>5	>5
K398.30	>5	>5
K398.31	>5	>5
K288.7	0.026	0.130
K288.8	0.013	0.131
K288.3	>5	0.136
K288.2	0.042	0.162
K288.1	0.881	5.115
K288.4	>5	>5
K288.5	>5	>5
K288.6	>5	>5
CC12.1	>5	0.051
CR3022	5.159	>5
DEN3	>5	>5

Neutralization: IC_{50}

Ab_ID	SARS-CoV-2-WIV1-RBD	SARS-CoV-2-RaTG13-RBD	SARS-CoV-2-pang17-RBD
K398.5	0.054	0.153	>5
K398.22	0.147	0.329	>5
K398.24	0.123	1.640	>5
K398.8	0.164	>5	>5
K398.25	0.330	1.521	0.114
K398.10	>5	>5	>5
K398.21	>5	>5	>5
K398.16	2.865	0.103	2.242
K398.18	2.227	>5	>5
K398.17	0.323	>5	0.408
K398.32	0.074	>5	>5
K288.7	0.302	>5	>5
K288.8	0.131	>5	>5
K288.3	0.827	>5	>5
K288.2	0.139	0.505	>5
CC12.1	>5	0.452	>5
DEN3	>5	>5	>5

Fig. S8. Binding and neutralization of vaccine-elicited monoclonal antibodies. Binding of mAbs isolated from immunized rhesus macaque B cells toward S-proteins. BLI binding response of isolated mAbs from K398 and K288 against SARS-CoV-2 S-protein, SARS-CoV-2 N terminal domain (NTD), SARS-CoV-2 RBD, SARS-CoV-2 RBD-SD1 (SD, sub-domain), SARS-CoV-2 RBD-SD1-SD2, SARS-CoV-1 S-protein, and SARS-CoV-1 RBD are shown in the left chart. The 50% pseudovirus neutralizing (IC_{50}) titers against SARS-CoV-1 and SARS-CoV-2 pseudoviruses are shown in the right chart. CC12.1, CR3022, and DEN3 were used as controls for the neutralization IC_{50} assays. The IC_{50} neutralization titers are shown for select cross-neutralizing mAbs with WIV1, RatG13, and pang17 RBD-SARS-CoV-2 chimeric pseudoviruses.

A

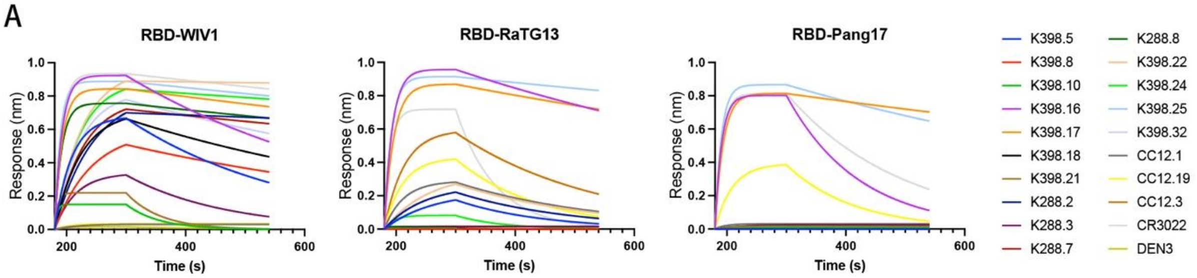

B

BLI Response (nm)	K398.5	K398.8	K398.10	K398.16	K398.17	K398.18	K398.21	K288.2	K288.3	K288.7	K288.8	K398.22	K398.24	K398.25	K398.32	CC12.1	CC12.19	CC12.3	CR3022	DEN3
Macaque-nAbs																				
RBD-WIV1	0.69	0.51	0.17	0.93	0.85	0.66	0.25	0.69	0.34	0.72	0.76	0.89	0.84	0.88	0.79	0.03	0.04	0.03	0.94	0.02
RBD-RaTG13	0.17	0.00	0.01	0.95	0.87	0.01	0.01	0.22	0.02	0.01	0.02	0.27	0.09	0.91	0.02	0.30	0.44	0.59	0.73	0.00
RBD-Pang17	0.00	0.00	0.01	0.82	0.82	0.00	0.02	0.01	0.02	0.02	0.00	0.00	0.02	0.86	0.02	0.03	0.40	0.02	0.84	0.01

Fig. S9. BLI binding of cross-reactive macaque antibodies with monomeric RBDs of ACE2-utilizing sarbecoviruses.

(A) BLI binding curves are shown for cross-reactive macaque antibodies with RBDs of WIV1, RatG13, and Pang17 sarbecoviruses. Human SARS-CoV-2 (CC12.1, CC12.19, and CC12.3), SARS-CoV-1 (CR3022) and unrelated Dengue (DEN3) specific antibodies were controls for the binding assay.

(B) Summary table showing peak of the BLI binding responses for each antibody with given monomeric RBD proteins. The BLI binding of the macaque antibodies is consistent with their cross-neutralizing activities with the corresponding sarbecoviruses.

Sequence characteristics

	HC				LC			
mAb ID	VH Gene	Num VSHMs	CDRH3 (AA)	CDRH3 length (AA)	VK/VL Gene	Num VSHMs	CDRH3 (AA)	CDRH3 length (AA)
**K398.25**	*IGHV1-105*01*	11	ATSSNTVLVAVATFEYFEF	19	*IGLV3-36*	8	QVWDSSSDHRI	11
**K288.5**	*IGHV1-138*01_S2593*	8	ARGGESGNYRGLYYFAY	17	*IGKV1-44*	29	QQVYSHPFT	9
**K398.9**	*IGHV1-138*02*	12	ARQEVVVVSASYYFDF	16	*IGLV2-32*	11	CSYAVSYSLML	11
**K398.29**	*IGHV1-138*02*	8	ARGSIAAGRFDF	12	*IGKV1-66*	9	QQYDNSPYS	9
**K398.6**	*IGHV1-NL_1*01_S2052*	19	VRSMGQLGRFDV	12	*IGKV1-32*	14	QQYDSLPFT	9
**K288.1**	*IGHV2-118*01_S2768*	7	ARVTMATGPVFENWLDV	17	*IGLV1-60*	15	AAWDDSLGGFI	11
**K288.4**	*IGHV2-69*01*	15	ARISRPGFSVSNVIDY	16	*IGLV2-38*	5	GSYRSGSTYI	10
**K398.23**	*IGHV3-187*02*	15	TRERPGGYCSGGVCYDVGYGLDS	23	*IGKV1-36*	17	LQSYTTPYT	9
**K398.1**	*IGHV3-50*01*	17	ARGACSGGVCNPDY	14	*IGLV5-69*	11	VIWHSGAWV	9
**K398.27**	*IGHV3-50*01*	16	ARANGGGYYSGLDS	14	*IGKV1-74*	10	QHNYGSPYT	9
**K398.30**	*IGHV3-50*01*	18	ARGTKPLDV	9	*IGKV1-69*	7	QQHDNSPYS	9
**K398.13**	*IGHV3-50*02_S9583*	17	GRGSGTIDY	9	*IGKV1-33*	5	QQRNTYPLT	9
**K398.19**	*IGHV3-50*02_S9583*	16	GRGSGTIDY	9	*IGLV1-81*	5	AAWDNSLNNGL	11
**K398.5**	*IGHV3-73*01*	14	SRIGITTFGLINRPFDL	17	*IGKV1-28*	15	LQYKNYPYT	9
**K398.22**	*IGHV3-73*01_S6942*	7	TRVSIFGQFIVATYFDY	17	*IGLV2-32*	4	CSYAGTVL	8
**K398.24**	*IGHV3-73*01_S6942*	14	ISRSLDSSGWSPRPLDY	17	*IGKV3S9*	17	QQYNSWWT	8
**K398.8**	*IGHV3-73*01_S6942*	12	CRVTILGMVIVRPFDY	16	*IGKV1-28*	8	LQYKSYPPLT	10
**K398.10**	*IGHV3-73*01_S6942*	12	TFPKGSWADYYGLGS	15	*IGKV1S15*	33	QQGYNYPWT	9
**K398.21**	*IGHV3-73*01_S6942*	12	SRPRPDIYGAYVYNGLDS	18	*IGKV1-43*	18	LRYGSYPWT	9
**K398.32**	*IGHV3-73*01_S6942*	12	TRPYLDWELDS	11	*IGLV2-32*	10	NA	-1
**K398.26**	*IGHV3-73*01_S6942*	7	TRVVTSPVGLVITNFDY	17	*IGLV2-32*	8	CSFAGPSYI	9
**K288.7**	*IGHV3-73*01_S6942*	6	TRVEITRMIVATNYFDY	17	*IGLV2-32*	11	CSYAASYTFYI	11
**K288.8**	*IGHV3-73*01_S6942*	14	TRVSIAGGLVVADWFDV	17	*IGLV2-23*	21	GSYAGSVL	8
**K288.3**	*IGHV3-73*01_S6942*	5	TRDRSQGAGGSIWTGYLSDGLDF	23	*IGKV1-28*	11	LQYKTYPRT	9
**K288.2**	*IGHV3-73*01_S6942*	7	TRPQSVTVFGVAATSYEAFDF	21	*IGKV3S9*	15	QQYTNWPLT	9
**K398.4**	*IGHV3-76*01*	12	SSTILPSRLDS	11	*IGLV5-83*	5	MIWHTNAYI	9
**K398.7**	*IGHV3-76*02_S4190*	17	AKGAANGFYRFDV	13	*IGKV1-66*	8	QQYDNFPPT	9
**K398.31**	*IGHV3-76*02_S4190*	14	AKGAANGFYRFDV	13	*IGKV1-66*	10	QQYNNSPPT	9
**K398.11**	*IGHV3-88*02_S9033*	8	ARYCSTIYCDARFDY	15	*IGLV2S9*	10	CSYRSGGTWV	10
**K398.28**	*IGHV3-NL_7*01_S0199*	44	VKGASLGDN	9	*IGKV1-74*	9	QHNYGTPCS	9
**K288.6**	*IGHV3-NL_7*01_S0199*	45	VKGASLGDN	9	*IGKV1-74*	21	QHGYGILT	8
**K398.18**	*IGHV4-149*01_S1992*	12	ARCEGATYPYWYFDV	15	*IGKV1-18*	10	QQGYNTPPT	9
**K398.12**	*IGHV4-79*02_S9501*	10	GRASRTADNY	10	*IGKV1S9*	12	QQHSSYPHT	9
**K398.16**	*IGHV5-15*01*	11	AKDGFYSTRWHYFGS	15	*IGLV2-38*	10	GSYRSGATFI	10
**K398.3**	*IGHV5-15*01_S9061*	17	ARVGGYRNIYDALDS	15	*IGLV4-97*	14	QTWATGIVL	9
**K398.14**	*IGHV5-15*01_S9061*	12	AKGYDYGYGFDY	12	*IGKV1-32*	16	QQYNSFPPT	9
**K398.15**	*IGHV5-15*01_S9061*	9	AKGEEFSVAAWLY	13	*IGKV1-32*	10	QQYNNLPYS	9
**K398.20**	*IGHV5-15*01_S9061*	14	AKGYDYGYGFDY	12	*IGLV1S6*	7	GAWDSSLSVWL	11
**K398.17**	*IGHV5-157*01*	12	AILTWNLWRFDV	12	*IGLV8-125*	16	LLYMGNGIWA	10
**K398.2**	*IGHV7-135*01*	10	ARSQIYGPSLDV	12	*IGLV3-16*	10	YSTYSSGNYGL	11

Fig. S10. Immunogenetic properties of mAbs isolated from two SARS-CoV-2 S-protein immunized rhesus macaques, K398 and K288. The heavy chain (HC) variable region (VH) and light chain (LC) variable region (VK/VL) gene usage, number of V region somatic hypermutations (SHM), CDR3 (third complementarity determining region) sequences, and lengths are shown for each mAb. The rhesus macaque (*Macaca mulatta*) germline database from (59) was used for all gene assignments.

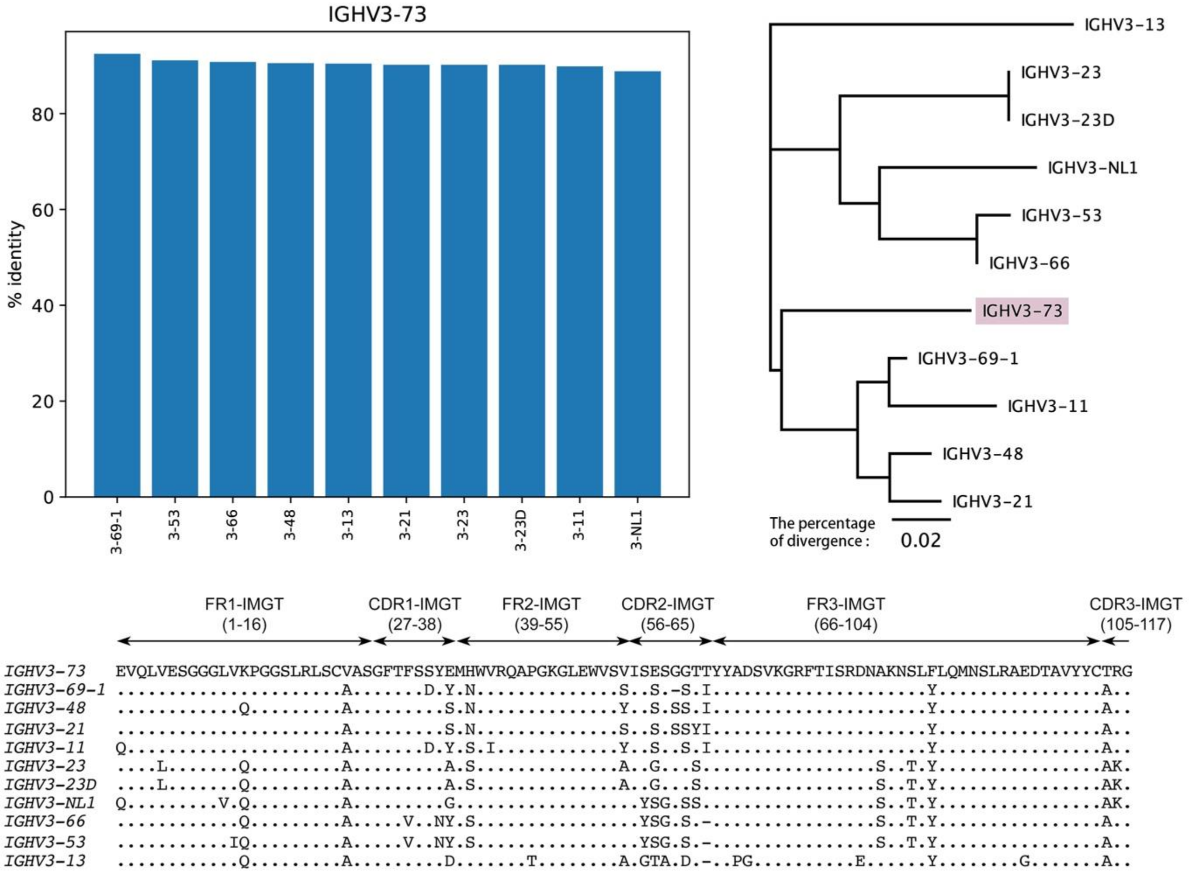

Fig. S11. Sequence similarity of macaque *IGHV3-73* germline with the closest human *IGHV* germline genes.

(A) The nucleotide sequence of the macaque *IGHV3-73* germline gene (utilized by macaque sarbecovirus cross-neutralizing Abs) was compared with the entire human *IGHV* germline gene database and the bar plot shows percent (%) identity with the 10 closest human germline genes (*IGHV3-69-1, IGHV3-53, IGHV3-66, IGHV3-48, IGHV3-13, IGHV3-21, IGHV3-23, IGHV3-23D, IGHV3-11* and *IGHV3-NL1*). The % identity for each human *IGHV* germline gene with macaque *IGHV3-73* germline is indicated. *IGHV3-69-1* is a pseudogene and is not expressed. The macaque *IGHV3-73* germline gene showed closest homology to the human *IGHV3-53* and *IGHV3-66* germline genes that are frequently enriched in human SARS-CoV-2 neutralizing antibodies.

(B) Phylogenetic relatedness of macaque *IGHV3-73* germline gene to the 10 closest human germline genes. The amino acid sequences of the macaque *IGHV3-73* germline gene and the closest human germline genes were used to generate the phylogenetic tree.

(C) The amino acid sequence alignment of macaque *IGHV3-73* germline V-gene with the 10 closest human germline genes is shown. The human germline genes are arranged in the order of their relatedness (at the amino acid level) to the macaque *IGHV3-73* germline gene.

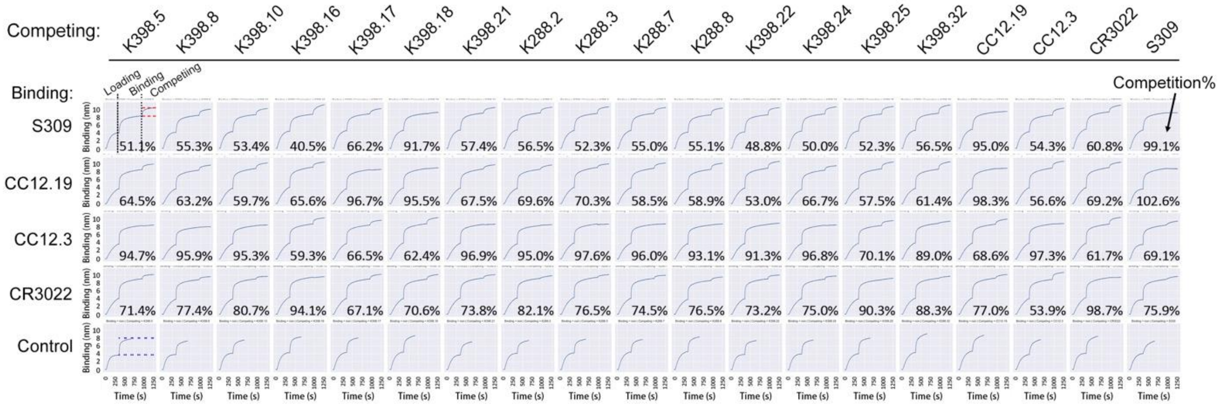

Fig. S12. Epitope mapping of macaque cross-neutralizing mAbs by BLI competition binning. The nAbs isolated from the SARS-CoV-2 S-protein immunized rhesus macaques were evaluated for epitope competition with human SARS-CoV-2 specific mAbs, CC12.19 and CC12.3, as well as cross-reactive S309 and CR3022 mAbs. His-tagged SARS-CoV-2 RBD protein was captured using anti-His biosensors and incubated with the indicated mAbs at a saturating concentration of 100µg/mL for 10 minutes and followed by an incubation for 5 minutes in the nAbs at concentration of 25µg/mL. BLI traces are shown for each binding. The binding inhibition % is calculated with the formula: percent (%) of inhibition in the BLI binding response = 1- (response in presence of the competitor antibody, indicated by red dashed line / response of the corresponding control antibody without the competitor antibody, indicated by blue line).

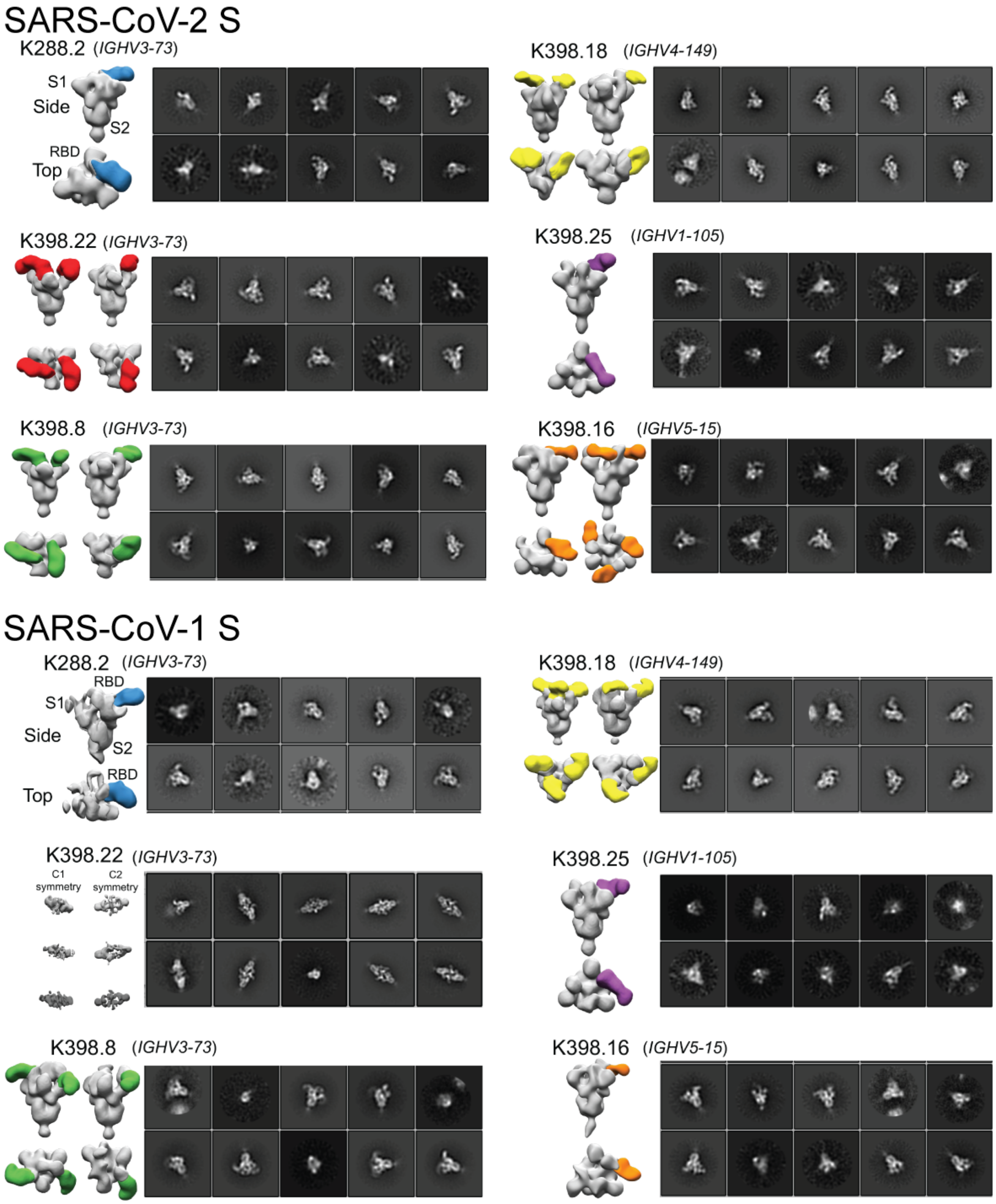

Fig. S13. Epitope mapping of sarbecovirus cross-neutralizing macaque mAbs bound to SARS-CoV-2 and SARS-CoV-1 S-proteins by negative stain electron microscopy. Electron microscopy (EM) 3D reconstructions are shown of sarbecovirus cross-neutralizing macaque antibodies K288.2 (blue), K398.22 (red), K398.8 (green), K398.18 (yellow), K398.25 (purple) and K398.16 (orange) in complex with SARS-CoV-2 (upper panel) and SARS-CoV-1 (lower panel) S-proteins. The *IGHV* gene usage for each macaque cross-neutralizing Ab is indicated. The EM 3D reconstructions of Fab and S-protein complexes were generated from nsEM 2D class average images shown for each mAb, under 3D reconstruction models. The S-protein S1 and S2 subunits and RBD are labelled for the representative cross-neutralizing Ab, K288.2.

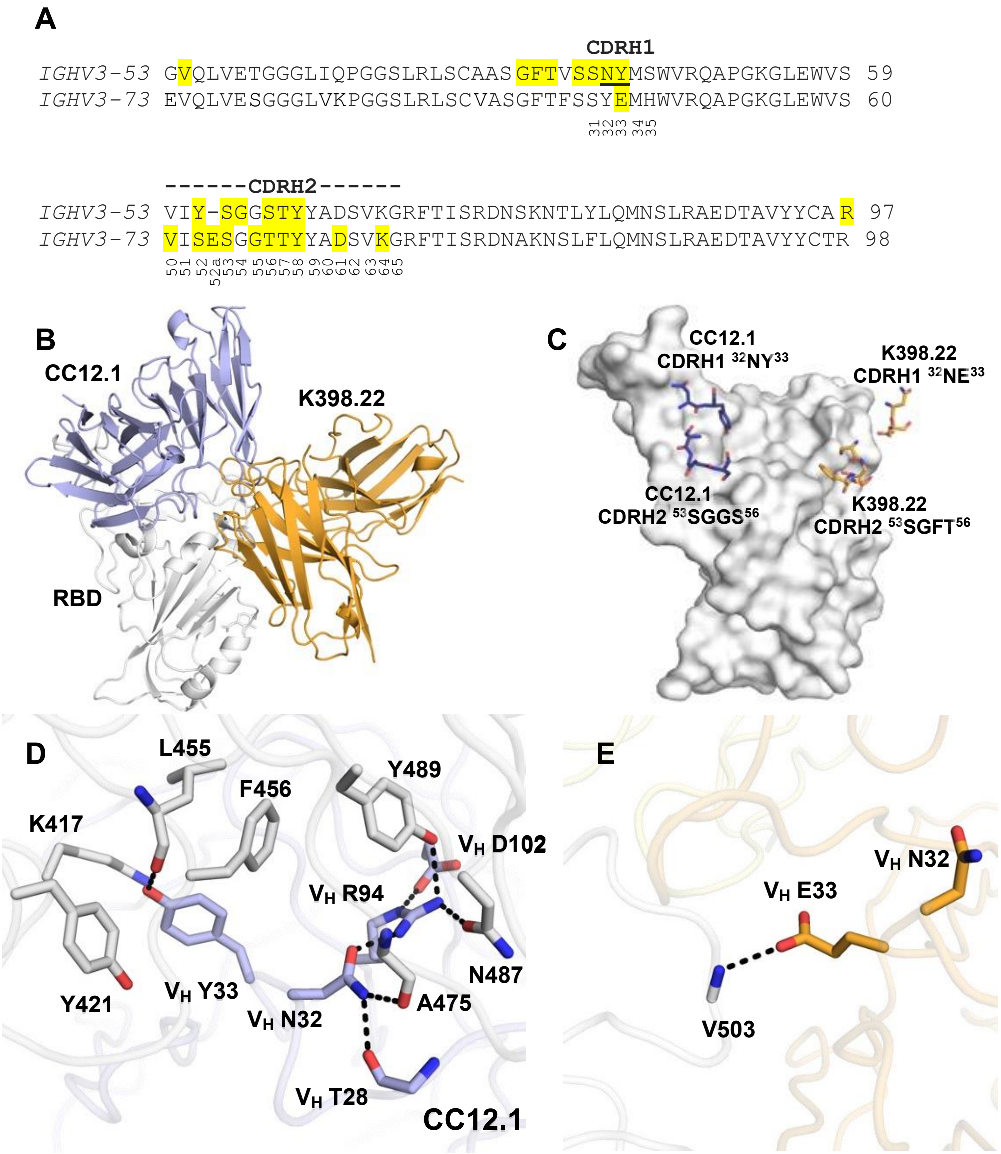

Fig. S14. Macaque *IGHV3-73* is closest to human *IGHV3-53* in sequence but differs in the critical RBD-interacting human germline 'NY' motif and adopts a completely different binding mode.

(A) Sequence alignment between the germlines (human *IGHV3-53* and macaque *IGHV3-73*) that encode shared antibody responses is shown. Paratope residues of *IGHV3-53* antibodies targeting SARS-CoV-2 in the canonical mode (22) as well as macaque *IGHV3-73* antibodies in this study are highlighted in yellow. Although the two germline sequences are similar (about 90% sequence identity), the critical RBD-interacting human IGHV3-53 'NY' motif (underlined) is replaced by 'YE' in macaque *IGHV3-73* and does not bind in the same mode.

(B) Macaque *IGHV3-73* and human *IGHV3-53* antibodies adopt different binding modes. K398.22 is used to represent the macaque *IGHV3-73* antibodies and CC12.1 for human IG*HV3-53* antibodies (PDB 6XC2). **(C)** Detailed interactions between SARS-CoV-2 RBD (white) and the CDRH1 'NY' motif of a human *IGHV3-53* antibody CC12.1 (PDB 6XC2) and CDRH1 'NE' motif of macaque *IGHV3-73* antibody K398.22 are shown.

(D and E) Interactions of corresponding (D) CC12.1 and (E) K398.22 CDRH1 residues with RBD are indicated. Hydrogen bonds and salt bridges are represented by black dashed lines.

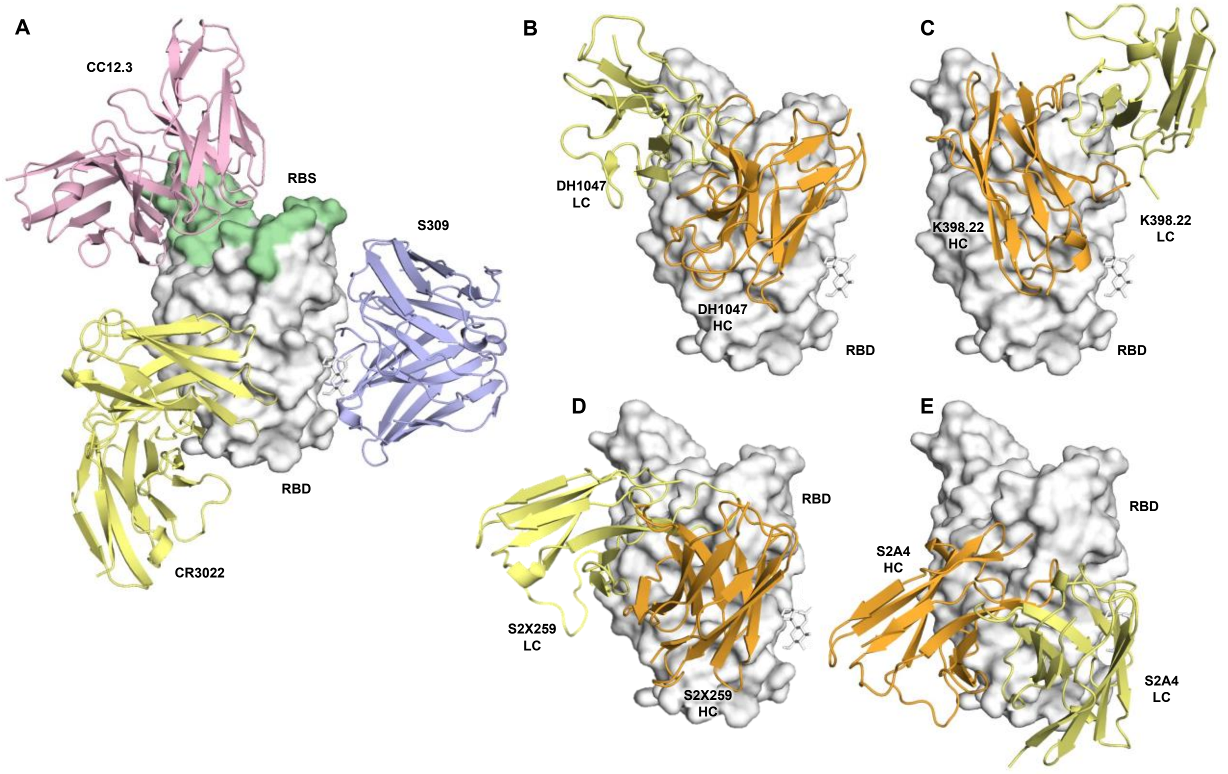

Fig. S15. Distinct binding modes of human and macaque RBD directed SARS-CoV-2 neutralizing antibodies. SARS-CoV-2 RBD is represented as a white surface. RBD molecules are in the same orientation in each panel. Antibodies are shown as cartoons in different colors. For clarity, only the variable domains of the antibodies are shown.

(**A**) Binding modes of representative human SARS-CoV-2 neutralizing antibodies (CC12.3: pink; CR3022: yellow; S309: blue) that bind to different epitopes on the RBD are shown. The N343 glycan is shown in stick representation. Each antibody interacts with the RBD in a different location and binding mode. The receptor binding site (RBS) on RBD is highlighted in green.

(**B to E**) Comparison between the binding of antibodies (B) DH1047, (C) K398.22, (D) S2X259, and (E) S2A4 are shown. For each antibody, the heavy chains are in orange and light chains in yellow. The heavy chains of three antibodies (DH1047, K398.22, and S2X259) target a similar region on the RBD, but in a different binding orientation. S2A4 binds lower down the RBD.

```
             320       330       340       350       360       370
              |         |         |         |         |         |
SARS-CoV-2-RBD VQPTESIVRFPNITNLCPFGEVFNATRFASVYAWNRKRISNCVADYSVLYNSA-S
RaTG13-RBD     ....D....................T........................T-.
Pang17-RBD     ....I...................SK........................T-.
SARS-CoV-1-RBD .V.SGDV..................K.P.....E..K.............T-F
WIV1-RBD       .A.SKEV..................T.P.....E................T-.
SHC014-RBD     .A.SKEV..................T.P.....E................T-.
BM4831-RBD     .T..TEV.......Q....N....I.S.P.....E.M..T...........SA.
BtKY72-RBD     .S.STEV............Q....SN.P.....E.L...D.....A.....SS.
RmYN02-RBD     IL.STEV........F...DK.......PN....Q.TK..D.I...T.....T-.
Rf1-RBD        .S.VTEV...........DK.......P.....E.TK..D.....T.F...T-.
Rs4081-RBD     .S..HEV........R...DK....S..PN....E.TK..D.....T.....T-.
Yun11-RBD      .S.STEVI.......R...DR....S..P.....E.TK..D.....T.....T-.

                    380       390       400       410       420
                     |         |         |         |         |
SARS-CoV-2-RBD FSTFKCYGVSPTKLNDLCFTNVYADSFVIRGDEVRQIAPGQTGKIADYNYKLPDD
RaTG13-RBD     ...........................T........................
Pang17-RBD     ...........................VK............V..........
SARS-CoV-1-RBD ..........A........S........VK..D.........V..........
WIV1-RBD       ..........A........S........VK..D.........V..........
SHC014-RBD     ..........A........S........VK..D.........V..........
BM4831-RBD     ....Q.............SS....Y..VK..D......A...V..........
BtKY72-RBD     ..................SS....Y..VK..D......A...V..........
RmYN02-RBD     ..........S..I.....S....T.L..FS.........E..V..........
Rf1-RBD        ....N......S..I.....S....T.L..FS....V......V..........
Rs4081-RBD     ..........S..I.....S....T.L..SS....V...E..V..........
Yun11-RBD      ..........S..I.....S....T.L..FS........E..V.........E

              430       440       450       460       470       480
               |         |         |         |         |
SARS-CoV-2-RBD FTGCVIAWNSNNLDSKVGGNYNYLYRLFRKSNLKPFERDISTEIYQAGSTPCNGV
RaTG13-RBD     ..........KHI.A.E...F.........A.................K....Q
Pang17-RBD     ..........VKQ.ALT....G.........K.....................Q
SARS-CoV-1-RBD .M...L...TR.I.ATST.....K..YL.HGK.R.......NVPFSPDGK..T-P
WIV1-RBD       .....L...TR.I.ATQT.....K..SL.HGK.R.......NVPFSPDGK..T-P
SHC014-RBD     .L...L...T.SK..STS........WV.R.K.N.Y...L.ND..SP.GQS.SA-
BM4831-RBD     .........T.S...SN----EFF..R..HGKI..YG..L.NVLFNPSGGT.SA-
BtKY72-RBD     .....L...T.SV...S.N--.FY.....HGKI..Y.....NVL.NSAGGT.SSI
RmYN02-RBD     .....L...TAQQ.IGS-----.F..SH.AVK.......L.SDE-----------
Rf1-RBD        .........TAKQ.VGS-----.F..SH.S.K.......L.S.E-----------
Rs4081-RBD     .........TAKQ.QGQ-----.Y..SS..TK.......LTSDE-----------
Yun11-RBD      .........TA.Q.RGQ-----.Y..SS..TK.......L.SDE-----------

                    490       500       510       520
                     |         |         |         |
SARS-CoV-2-RBD EGFNCYFPLQSYGFQPTNGVGYQPYRVVVLSFELLHAPATVCGP
RaTG13-RBD     T.L...Y..YR...Y..D...H.............N........
Pang17-RBD     V.L...Y..ER...H..T..N...F..........NG.......
SARS-CoV-1-RBD PAL...W..ND...YT.T.I...............N........
WIV1-RBD       PA....W..ND...YI...I...............N........
SHC014-RBD     V.P...N..RP...FT.A...H.............N........
BM4831-RBD     ..L...K..A....TQSS.I.F.............N........
BtKY72-RBD     SQLG..E..K....T..V.................N........
RmYN02-RBD     ---.GVRT.ST.D.N.NVPLD..AT..........N........
Rf1-RBD        ---.GVRT.ST.D.NQNVPLE..AT..........N........
Rs4081-RBD     ---.GVRT.ST.D.Y.NVPIE..AT..........N........
Yun11-RBD      ---.GVRT.ST.D.Y.SVPLE..AT..........N........
```

Fig. S16. Conservation of the RBD broadly neutralizing Ab epitope recognized by macaque *IGHV3-73* encoded cross-nAbs. Amino acid sequence alignment of sarbecovirus RBD regions from 12 major lineages are shown, as indicated on the left. Sequence conservation of the epitope residues (highlighted in gray) in SARS-related strains recognized by macaque *IGHV3-73* encoded broadly neutralizing Abs.

Table S1. X-ray data collection and refinement statistics

**Data collection**		
	Fab K288.2 + RBD	Fab K398.22 + RBD
Beamline	SSRL 12-1	APS23 ID-D
Wavelength (Å)	0.97946	1.03321
Space group	$P\ 2_1\ 2_1\ 2_1$	$C\ 2\ 2\ 2_1$
Unit cell		
a, b, c (Å)	72.5, 88.6, 131.5	68.6, 277.3, 91.4
α, β, γ (°)	90, 90, 90	90, 90, 90
Resolution (Å) [a]	50.0–2.33 (2.37–2.33)	46.2–1.95 (2.00–1.95)
Unique	37,765 (2,957)	63,980 (6,317)
Redundancy [a]	7.3 (5.4)	13.2 (13.7)
Completeness	97.7 (85.8)	99.9 (99.8)
$\langle I/\sigma_I \rangle$ [a]	18.9 (1.1)	14.5 (2.2)
R_{sym}[b] (%) [a]	14.3 (>100)	8.6 (>100)
R_{pim}[b] (%) [a]	5.6 (46.4)	2.5 (41.7)
$CC_{1/2}$[c] (%) [a]	96.5 (53.3)	99.9 (93.6)
**Refinement statistics**		
Resolution (Å)	36.3–2.33	45.7–1.95
Reflections	35,760	63,811
Reflections (test)	1,785	3,294
R_{cryst}[d] / R_{free}[e] (%)	18.5/23.1	22.3/24.1
No. of atoms	5,065	5,101
Macromolecul	4,937	4,743
Glycans	0	53
Solvent	113	305
Average *B*-value	58	53
Macromolecul	58	54
Glycans	N/A	32
Solvent	54	53
Wilson *B*-value	49	43
**RMSD from ideal geometry**		
Bond length (Å)	0.003	0.012
Bond angle (°)	0.69	1.45
**Ramachandran statistics (%)**		
Favored	96.5	96.4
Outliers	0.16	0.16
**PDB code**	7TP3	7TP4

[a] Numbers in parentheses refer to the highest resolution shell.

[b] $R_{sym} = \Sigma_{hkl} \Sigma_i \mid I_{hkl,i} - \langle I_{hkl} \rangle \mid / \Sigma_{hkl} \Sigma_i I_{hkl,i}$ and $R_{pim} = \Sigma_{hkl} (1/(n-1))^{1/2} \Sigma_i \mid I_{hkl,i} - \langle I_{hkl} \rangle \mid / \Sigma_{hkl} \Sigma_i I_{hkl,i}$, where $I_{hkl,i}$ is the scaled intensity of the ith measurement of reflection h, k, l, $\langle I_{hkl} \rangle$ is the average intensity for that reflection, and *n* is the redundancy.

[c] $CC_{1/2}$ = Pearson correlation coefficient between two random half datasets.

[d] $R_{cryst} = \Sigma_{hkl} \mid F_o - F_c \mid / \Sigma_{hkl} \mid F_o \mid \times 100$, where F_o and F_c are the observed and calculated structure factors, respectively.

[e] R_{free} was calculated as for R_{cryst}, but on a test set comprising 5% of the data excluded from refinement.

[f] Calculated by PHENIX (92).

Table S2. Hydrogen bonds identified at the antibody-RBD interface using the PISA (Proteins, Interfaces, Structures and Assemblies) program.

SARS-CoV-2 RBD	Distance	K288.2
ASP405[OD1]	2.9	VH:THR57[OG1]
ASP405[OD1]	3.5	VH:THR57[N]
ASP405[OD1]	3.0	VH:SER52[OG]
THR500[O]	2.9	VH:TYR112[N]
THR500[OG1]	3.5	VH:SER111[OG]
GLN498[NE2]	3.8	VH:SER111[OG]
ASN501[ND2]	3.0	VH:THR110[O]
ASN501[ND2]	3.4	VH:SER111[OG]
GLY502[N]	2.9	VH:THR110[O]
VAL503[N]	2.9	VH:GLU33[OE2]
GLN506[OE1]	2.9	VL:TRP94[NE1]
ASN439[ND2]	3.2	VL:THR92[O]
**SARS-CoV-2 RBD**	**Distance [Å]**	**K398.22**
ASP405[OD1]	2.9	VH:SER52[OG]
ASP405[OD2]	2.5	VH:SER54[OG]
ASP405[OD1]	3.4	VH:THR57[N]
ASP405[OD1]	2.6	VH:THR57[OG1]
SER373[OG]	2.7	VH:LYS65[NZ]
THR500[O]	3.2	VH:ILE106[N]
VAL503[N]	2.9	VH:GLU33[OE1]
TYR505[OH]	2.8	VH:GLU53[OE2]
ASP405[N]	3.1	VH:THR57[OG1]
TYR508[OH]	2.7	VH:GLU59[OE2]
ASN501[ND2]	3.0	VH:GLN104[O]
GLY502[N]	3.0	VH:GLN104[O]
GLN506[NE2]	3.0	VL:GLY95[O]
